# Associations between short-term PM_2.5_ exposure and daily hospital admissions for circulatory system diseases in Ganzhou, China: A time series study

**DOI:** 10.3389/fpubh.2023.1134516

**Published:** 2023-03-09

**Authors:** Xiaojie You, Xiuyu Cao, You Guo, Dongming Wang, Weihong Qiu, Chuanfei Zhou, Min Zhou, Weihong Chen, Xiaokang Zhang

**Affiliations:** ^1^Department of Occupational and Environmental Health, School of Public Health, Tongji Medical College, Huazhong University of Science and Technology, Wuhan, Hubei, China; ^2^Key Laboratory of Environment and Health, Ministry of Education and Ministry of Environmental Protection, and State Key Laboratory of Environmental Health (Incubating), School of Public Health, Tongji Medical College, Huazhong University of Science and Technology, Wuhan, Hubei, China; ^3^First Affiliated Hospital, Gannan Medical University, Ganzhou, China; ^4^Key Laboratory of Prevention and Treatment of Cardiovascular and Cerebrovascular Diseases, Ministry of Education, Gannan Medical University, Ganzhou, China; ^5^School of Public Health and Health Management, Gannan Medical University, Ganzhou, China

**Keywords:** circulatory system diseases, PM_2.5_, hospital admissions, air pollution, generalized additive model (GAM), time series study

## Abstract

**Objective:**

Previous epidemiological studies have shown that both long-term and short-term exposure to fine particulate matters (PM_2.5_) were associated with the morbidity and mortality of circulatory system diseases (CSD). However, the impact of PM_2.5_ on CSD remains inconclusive. This study aimed to investigate the associations between PM_2.5_ and circulatory system diseases in Ganzhou.

**Methods:**

We conducted this time series study to explore the association between ambient PM_2.5_ exposure and daily hospital admissions for CSD from 2016 to 2020 in Ganzhou by using generalized additive models (GAMs). Stratified analyses were also performed by gender, age, and season.

**Results:**

Based on 201,799 hospitalized cases, significant and positive associations were found between short-term PM2.5 exposure and hospital admissions for CSD, including total CSD, hypertension, coronary heart disease (CHD), cerebrovascular disease (CEVD), heart failure (HF), and arrhythmia. Each 10 μg/m^3^ increase in PM_2.5_ concentrations was associated with a 2.588% (95% confidence interval [CI], 1.161%–4.035%), 2.773% (95% CI, 1.246%–4.324%), 2.865% (95% CI, 0.786%–4.893%), 1.691% (95% CI, 0.239%–3.165%), 4.173% (95% CI, 1.988%–6.404%) and 1.496% (95% CI, 0.030%–2.983%) increment in hospitalizations for total CSD, hypertension, CHD, CEVD, HF, and arrhythmia, respectively. As PM_2.5_ concentrations rise, the hospitalizations for arrhythmia showed a slow upward trend, while other CSD increased sharply at high PM_2.5_ levels. In subgroup analyses, the impacts of PM_2.5_ on hospitalizations for CSD were not materially changed, although the females had higher risks of hypertension, HF, and arrhythmia. The relationships between PM_2.5_ exposure and hospitalizations for CSD were more significant among individuals aged ≤65 years, except for arrhythmia. PM_2.5_ had stronger effects on total CSD, hypertension, CEVD, HF, and arrhythmia during cold seasons.

**Conclusion:**

PM_2.5_ exposure was positively associated with daily hospital admissions for CSD, which might provide informative insight on adverse effects of PM_2.5_.

## 1. Introduction

The development of modern industrialization has made air pollution one of the leading public health concerns worldwide (1). According to the latest Global Burden of Disease (GBD) Survey, ambient air pollution could be responsible for 6.7 million deaths in 2019 (2). Accumulating studies have shown that exposure to air pollutants, whether both short- or long-term, may damage human health on multiple levels (3).

PM_2.5_, named particulate matter with aerodynamic diameter below 2.5 μm, is considered the most sensitive indicator of air quality (4). Ambient PM_2.5_ mainly comes from natural conditions (volcanic eruptions and dust storms) and human activities such as industrial production, and traffic exhaust emissions (5). PM_2.5_ has become an environmental problem and has attracted global public health concerns because of its adverse effects on multiple organs (6). Previous studies suggested that PM_2.5_ exposure might lead to increased risks of respiratory diseases (7), circulatory system diseases (CSD) (8), neurological diseases (9), and metabolic diseases (10). In fact, the impact of PM_2.5_ on CSD has been extensively reported in previous studies (11–13). However, epidemiologic studies regarding the relationships of short-term exposure to PM_2.5_ and CSD remain inconclusive. According to a national study containing 379,133 participants, there was a 0.12% (95% CI, 0.001–0.25%) elevation in cardiovascular disease mortality with a 10-μg/m^3^ increase in PM_2.5_ levels on the same day (14). However, a study (15) including over 286 million hospitalizations in England and Wales found little evidence of PM_2.5_ exposure with increased risk of cardiovascular admissions, and even in many cases, PM_2.5_ was related to decrease risks of cardiovascular hospitalization. More studies are warranted to evaluate the impacts of short-term PM_2.5_ exposure on CSD risks.

Ganzhou, a city with 9.8402 million population in 2021 and located in southern China, enjoys a typical subtropical monsoon climate. In recent years, great measures were conducted to reduce urban ambient pollution in China and the air quality of Ganzhou has gradually improved in last 10 years. In 2021, the mean concentration of PM_2.5_ in Ganzhou was 23 μg/m^3^, 34.3% lower than the that (35 μg/m^3^) in 168 Chinese cities in the same year. To evaluate potential effects of PM_2.5_ on the onset of CSD in Ganzhou is also helpful to understand adverse health effects caused by relatively low levels of PM_2.5_ in China_._

In this study, daily concentrations of air pollutants in Ganzhou were collected from China National Environmental Monitoring Center (CNEMC), and hospital admission data for CSD from 2016 to 2020 were extracted from the biggest hospital in Ganzhou. This time-series analysis was conducted to evaluate the relationships of ambient PM_2.5_ exposure with daily hospitalizations for CSD. The effects of co-exposure to other air pollutants on above relationships were also analyzed.

## 2. Materials and methods

### 2.1. Daily hospital admissions data

Daily hospital admissions data of CSD from Jan.1, 2016 to Dec.31, 2020 were extracted from the hospital's admission case registry system in the biggest hospital of Ganzhou. The patient information included gender, age, residential address, date of admission, and principal diagnosis. The CSD in present study were encoded according to *the 10th version of the International Classification of Diseases* (ICD-10) as follows: total circulatory disease (I00-I99), hypertension (I10-I15), coronary heart disease (CHD, I20-I25), cerebrovascular diseases (CEVD, I60-I69), heart failure (HF, I50), and arrhythmia (I47-I49).

### 2.2. Ambient air pollutants and meteorological data

The 24-h average concentrations of PM_2.5_, NO_2_, PM_10_, SO_2_, and CO, and the maximum 8-h mean concentrations of O_3_ from January 1, 2016 to December 31, 2020 were obtained from the National Real-Time Air Quality Monitoring Data Publishing Platform developed by CNEMC (http://www.cnemc.cn/). Daily average meteorological data including daily average temperature, relative humidity, and wind speed during the 5 years were obtained from the National Meteorological Information Center (http://data.cma.cn/). Present study did not involve/include any personally identifiable information and Institutional Review Board approval was not applicable.

### 2.3. Statistical analysis

Descriptive statistical analyses were performed to reveal the features of daily circulatory hospital admissions, ambient air pollutants and meteorological factors from January 1, 2016 to December 31, 2020. Spearman's rank correlation test was used to evaluate the bivariate associations between air pollutants and meteorological variables. To address the associations between PM_2.5_ and hospitalization for CSD, the quasi-Poisson regression method in generalized additive models (GAMs) based on time series was applied. In this model, spline smoothing functions of time trend, daily mean temperature and relative humidity were introduced into the GAMs to exclude the potential confounding effects of long-term time trend and meteorological variables. Besides, the day of the week (DOW) and holiday were also considered as potential confounders. The model finally used was as follows:


$$\begin{array}{l} \;Log\left[E\left(Yt\right)\right]=\;\alpha +\beta Xt+s\left(time,\;df=14\;per\;year\right)\\ +s\left(Temp,\;df=3\right)+s\left(RH,\;df=3\right)\\ +as.factor\left(DOW\right)+as.factor\left(Holiday\right)\; \end{array}$$

Where *t* is the day of observation; *Yt* denotes daily count of hospital admissions for CSD at day t; *Xt* is the daily mean concentration of PM_2.5_ at day t; β is the regression coefficient which represents the log-relative rate of hospital admissions for CSD with a 10 μg/m^3^ increase of PM_2.5_ concentration; *DOW* means the day of the week; *df* is degree of freedom whose value is determined based on the Quasi-Akaike information criterion (QAIC); α is the model intercept.

It is universally accepted that ambient air pollutants have persistent and hysteresis effects on health outcomes (16). We considered both single-day lags (lag0–lag14) and multiday lags (lag01–lag014) to evaluate delayed effects of PM_2.5_. Lag0 refers to the impact of PM_2.5_ on hospitalizations for CSD on the same day and Lag01 shows the effect of PM_2.5_ in the current and the previous days. Additionally, stratified analyses were performed to determine whether the associations differed by age (≤65 and >65 years old), gender, season (May-October, warm season; December-April, cold season).

To exclude the potential confounding effect of other pollutants, a series of co-pollutant models were developed in addition to the single-pollutant model. Notably, to avoid collinearity, pollutants with correlation coefficients >0.6 were not incorporated into co-pollutant models simultaneously.

In the sensitivity analysis, we adjust the dfs of calendar time to 7 per year to test the stability of the associations, according to relevant studies (17–19). In addition, previous studies have shown that COVID-19 might have impacts on circulatory system (20). In this study, in order to control the impact of COVID-19 on the associations, we also included the occurrence of COVID-19 in the model for sensitivity analysis.

All results were given as percent changes and 95% confidence intervals (CIs) in daily hospitalizations for CSD per 10 μg/m^3^ increment in PM_2.5_ levels. All statistical analyses were performed in R version 4.1.2 with the “mgcv” and “tsModel” packages. *P*-value < 0.05 was considered as statistically significant (2-sided).

## 3. Results

The descriptive statistics of daily air pollutants, meteorological variables, and hospitalizations for CSD during the 5 years were shown in Table 1. A total of 201,799 hospitalizations for CSD (daily average: 110 hospital admissions) in Ganzhou were included in the analysis. Among all the records, 50.5% were older than 65 years old, and 56.8% were males. As for disease subtypes, hypertension accounted for 47.0% of total CSD, followed by cerebrovascular diseases (20.9%), coronary heart disease (14.2%), heart failure (10.7%) and arrhythmia (7.2%).

**Table 1:** Characteristics of meteorological variables, ambient air pollutants, and hospital admissions in Ganzhou (2016–2020).

	***N* (%)**	**Mean ±Sd**	**Min**	**P25**	**P50**	**P75**	**Max**
**Meteorological factors**							
Temperature (°C)	–	20.54 ± 8.09	1	13.69	21.7	27.79	33.5
Relative Humidity (%)	–	75.21 ± 12.00	35.5	66.3	75.5	84.5	99
**Air pollutants**							
PM_2.5_ (μg/m3)	–	37.38 ± 20.8	6	23	33	47	184
PM_10_ (μg/m3)	–	60.06 ± 33.60	11	36	52	75	246
NO_2_ (μg/m3)	–	22.75 ± 12.60	4	14	19	28	84
SO_2_ (μg/m3)	–	18.73 ± 11.18	2	11	16	23	73
CO (mg/m3)	–	1.24 ± 0.32	0.6	1	1.2	1.43	2.9
O_3_ (μg/m3)	–	90.52 ± 39.09	7	62.5	88	116	224
**Hospital admissions**							
Total	201,799 (100)	110.0 ± 45.10	18	77	103	139	308
Male	114,671 (56.8)	62.76 ± 26.70	9	44	58	79	177
Female	87,101 (43.2)	47.67 ± 19.90	6	33	45	60	131
≤ 65 years old	99,921 (49.5)	54.69 ± 24.30	6	37	51	69	150
>65 years old	101,878 (50.5)	55.76 ± 23.10	7	38	52	71	160
Cold	100,711 (49.91)	111.0 ± 44.98	18	79	105	138	308
Warm	101,088 (50.09)	109.0 ± 45.17	25	77	102	139	281
**CSD**							
Total	201,799 (100)	110.0 ± 45.10	18	77	103	139	308
Hypertension	94,844 (47.0)	51.91 ± 22.00	8	36	49	65	155
CHD	28,597 (14.2)	15.00 ± 7.36	0	10	15	20	49
CEVD	42,120 (20.9)	23.05 ± 8.79	2	16	22	29	57
HF	21,636 (10.7)	11.84 ± 6.73	0	7	10	16	40
Arrhythmia	14,602 (7.2)	7.99 ± 4.93	0	4	7	11	29

N, number; Sd, standard deviation; P, percentile; CSD, circulatory system diseases; CEVD, Cerebrovascular Disease; CHD, Coronary Heart Disease; HF, Heart Failure.

The average daily concentrations of PM_2.5_, PM_10_, NO_2_, SO_2_ and CO during research period were 37.38 μg/m^3^, 60.06 μg/m^3^, 22.75 μg/m^3^, 18.73 μg/m^3^, 1.24 mg/m^3^, respectively, while the 8-hour average concentration of O_3_ was 90.52 μg/m^3^. On average, the daily temperature was 20.54°C and the relative humidity was 75.21 %.

As shown in the time series plots, the levels of PM_2.5_ in Ganzhou decreased slowly and gradually during the study period (Supplementary Figure 1). The levels of PM_10_, NO_2_, SO_2_ and CO also showed similar trends, except that O_3_ levels increased. During the same period, hospital admissions for total CSD and five specific subtypes fluctuated and rose steadily (Supplementary Figure 2).

**Figure 1:**
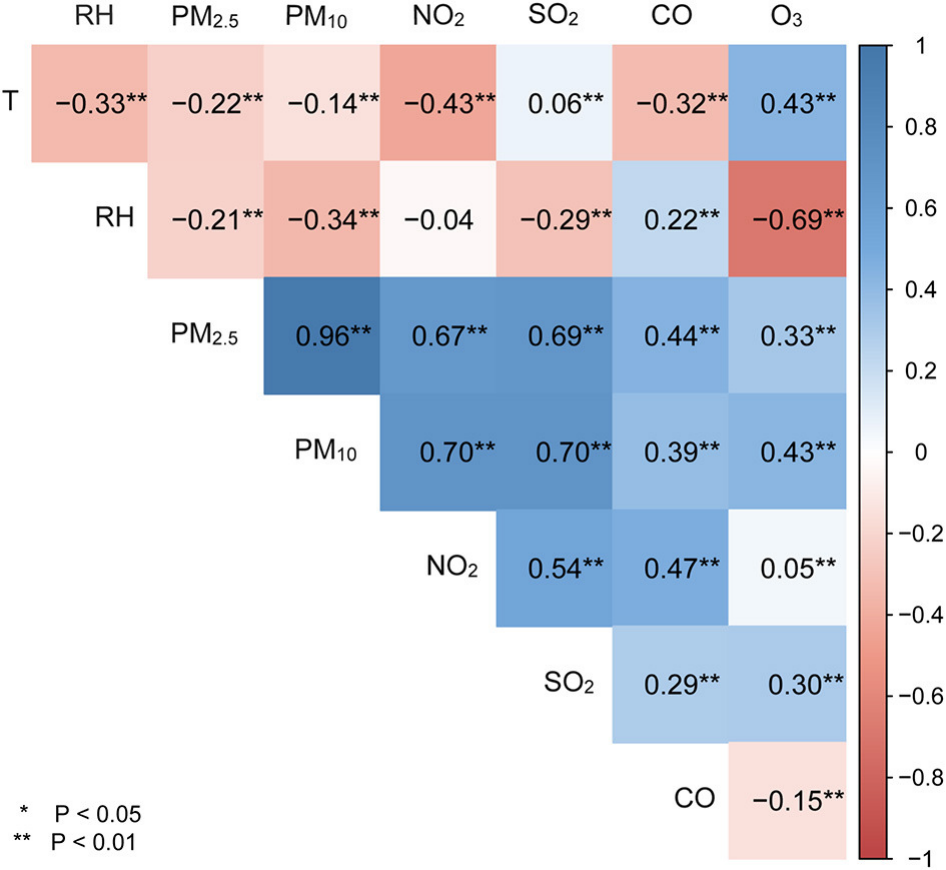
Spearman's rank correlations of meteorological variables with ambient air pollutants. T represents temperature; RH denotes relative humanity; *means *P* < 0.05, **means *P* < 0.01.

The Spearman's correlation coefficients for exposure variables were given in Figure 1. PM_2.5_ was highly correlated with PM_10_ and SO_2_, and NO_2_ (*r* > 0.6 and *P* < 0.05), moderately correlated with CO and O_3_ (*r* = 0.44 and 0.33, respectively, *P* < 0.05). However, PM_2.5_ was negatively correlated with relative humidity (*r* = −0.21, *P* < 0.05) and temperature (*r* = −0.22, *P* < 0.05).

**Figure 2:**
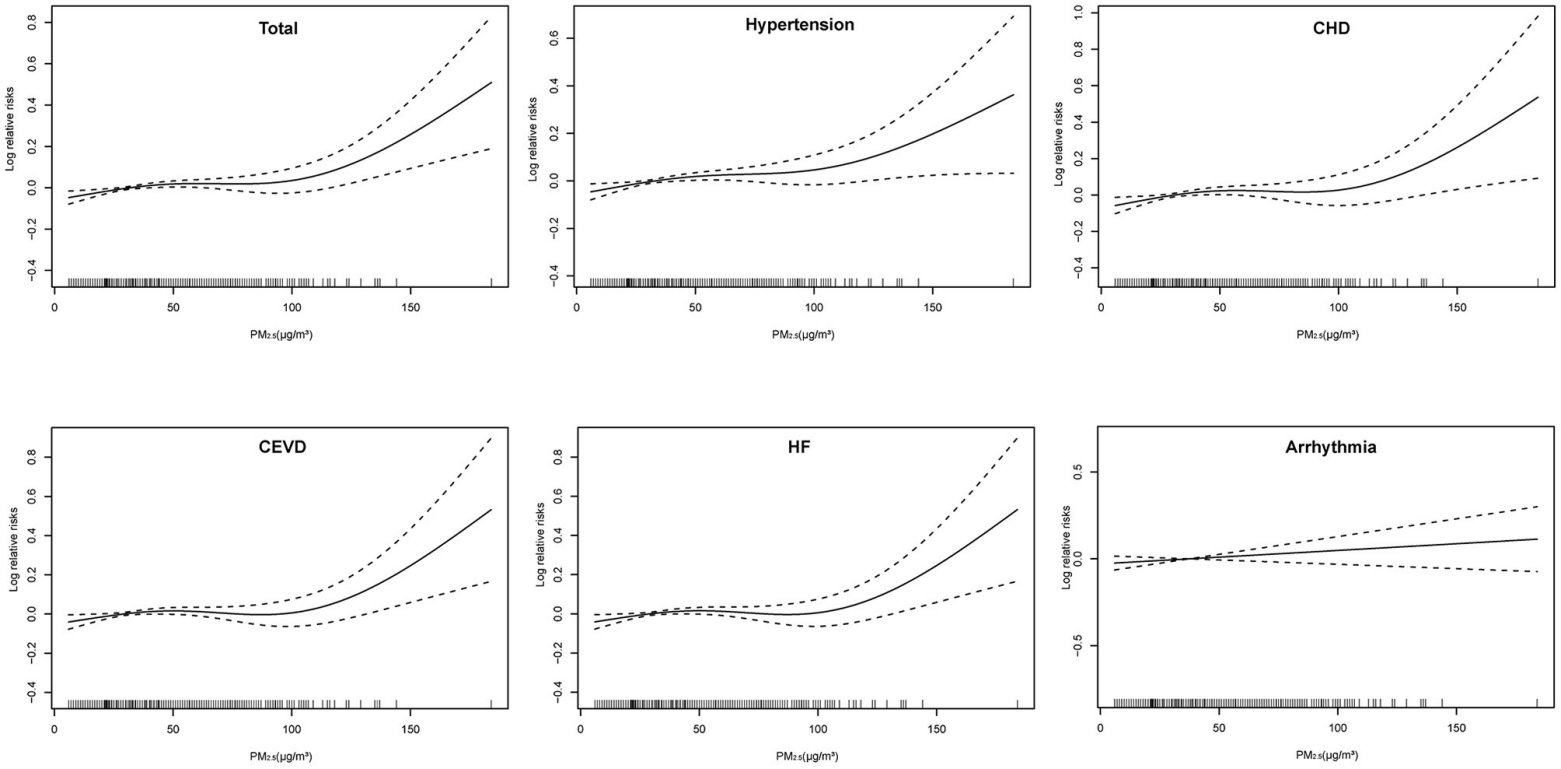
Exposure-response relationships of PM_2.5_ concentrations with daily hospital admissions for total and cause-specific circulatory system diseases. CEVD, Cerebrovascular Disease; CHD, Coronary Heart Disease; HF, Heart Failure. The concentration response curve is the solid line and the 95% CI is presented by the dotted line.

Positive linear exposure-response relationships between PM_2.5_ concentrations and daily hospitalizations for total and cause-specific CSD were observed (Figure 2). Hospitalizations for total CSD, hypertension, CHD, CEVD, and HF increased rapidly at high levels of PM_2.5_, except for arrhythmia, which showed a slowly linear rise. In single-pollutant models, significantly positive associations were observed between PM_2.5_ levels and hospital admissions for studied CSD in both single-day (lag1–lag14) and cumulative-day (lag01–lag014) lag structures (Table 2). The largest single day effect of PM_2.5_ was at lag6 for total CSD, hypertension, CHD, lag4 for CEVD, and lag1 for HF and arrhythmia. The greatest cumulative day effect for total CSD, hypertension, CHD and HF were observed at lag014. The effect of PM_2.5_ on hospitalizations for CEVD peaked at lag011. Every 10 μg/m^3^ increment of ambient PM_2.5_ concentrations was associated with a 2.588% [95% confidence interval (CI), 1.161–4.035%], 2.773% (95% CI, 1.246–4.324%), 2.865% (95% CI, 0.786–4.893%), 1.691% (95% CI, 0.239–3.165%), 4.173% (95% CI, 1.988–6.404%) and 1.496% (95% CI, 0.030–2.983%) increment in hospitalizations for total CSD, hypertension, CHD, CEVD, HF, and arrhythmia, respectively.

**Table 2:** Percent changes and 95% confidence intervals (CIs) of daily hospital admissions for total and cause-specific circulatory system diseases by each 10 μg/m^3^ increase of PM_2.5_ concentrations at different lag structures in the single-pollutant model.

**Lag Type**	**Lag day**	**Total CSD**	**Hypertension**	**CHD**	**CEVD**	**HF**	**Arrhythmia**
Single-lag	0	0.245 (−0.495, 0.990)	0.134 (−0.660, 0.935)	0.402 (−0.632, 1.446)	−0.125 (−0.972, 0.730)	**1.436 (0.326, 2.558)**	0.804 (−0.541, 2.168)
	1	**0.880 (0.157, 1.609)**	0.758 (−0.017, 1.538)	0.840 (−0.180, 1.870)	0.443 (−0.380, 1.274)	**2.277 (1.172, 3.393)**	**1.638 (0.320, 2.972)**
	2	0.560 (−0.147, 1.272)	0.586 (−0.170, 1.348)	0.349 (−0.649, 1.356)	0.279 (−0.526, 1.091)	**1.655 (0.571, 2.750)**	0.557 (−0.736, 1.866)
	3	0.305 (−0.394, 1.01)	0.442 (−0.308, 1.198)	−0.084 (−1.072, 0.914)	0.132 (−0.667, 0.937)	0.898 (−0.167, 1.974)	0.359 (−0.918, 1.653)
	4	**0.691 (0.001, 1.386)**	**0.822 (0.080, 1.569)**	0.378 (−0.600, 1.365)	**0.873 (0.083, 1.670)**	0.462 (−0.585, 1.520)	0.562 (−0.700, 1.84)
	5	**0.935 (0.250, 1.624)**	**1.134 (0.397, 1.875)**	0.667 (−0.304, 1.647)	**0.858 (0.072, 1.651)**	**1.079 (0.042, 2.127)**	0.395 (−0.859, 1.664)
	6	**1.099 (0.421, 1.782)**	**1.129 (0.399, 1.864)**	**1.185 (0.223, 2.156)**	0.767 (−0.015, 1.555)	**1.934 (0.904, 2.975)**	0.774 (−0.474, 2.038)
	7	**0.912 (0.233, 1.595)**	**1.084 (0.354, 1.820)**	0.804 (−0.159, 1.776)	0.692 (−0.088, 1.479)	**1.336 (0.305, 2.378)**	0.206 (−1.040, 1.468)
	8	**0.711 (0.029, 1.398)**	**0.925 (0.190, 1.665)**	0.597 (−0.370, 1.573)	0.555 (−0.229, 1.345)	0.747 (−0.282, 1.788)	0.114 (−1.133, 1.376)
	9	**0.747 (0.070, 1.429)**	**0.973 (0.244, 1.707)**	0.735 (−0.225, 1.704)	0.558 (−0.222, 1.344)	0.562 (−0.459, 1.594)	0.254 (−0.981, 1.505)
	10	**0.689 (0.009, 1.374)**	0.690 (−0.043, 1.429)	**1.060 (0.095, 2.034)**	0.784 (0.000, 1.575)	0.423 (−0.597, 1.453)	0.252 (−0.986, 1.506)
	11	0.488 (−0.196, 1.176)	0.584 (−0.153, 1.327)	0.965 (−0.007, 1.946)	0.179 (−0.607, 0.971)	0.228 (−0.795, 1.261)	0.347 (−0.899, 1.608)
	12	0.504 (−0.184, 1.196)	0.475 (−0.267, 1.223)	**1.277 (0.299, 2.266)**	0.029 (−0.760, 0.824)	0.528 (−0.501, 1.567)	0.708 (−0.545, 1.978)
	13	0.219 (−0.470, 0.913)	0.246 (−0.498, 0.996)	0.720 (−0.261, 1.711)	−0.252 (−1.041, 0.544)	0.533 (−0.498, 1.575)	0.177 (−1.079, 1.448)
	14	0.223 (−0.466, 0.916)	0.207 (−0.537, 0.957)	0.473 (−0.506, 1.461)	−0.24 (−1.030, 0.556)	0.522 (−0.511, 1.566)	0.958 (−0.304, 2.236)
Cumulative-lag	01	0.697 (−0.111, 1.512)	0.555 (−0.310, 1.427)	0.758 (−0.376, 1.905)	0.198 (−0.723, 1.128)	**2.305 (1.077, 3.547)**	**1.496 (0.030, 2.983)**
	02	0.820 (−0.051, 1.698)	0.717 (−0.211, 1.655)	0.766 (−0.453, 1.999)	0.290 (−0.697, 1.287)	**2.632 (1.301, 3.981)**	1.441 (−0.134, 3.041)
	03	0.836 (−0.091, 1.772)	0.798 (−0.190, 1.796)	0.631 (−0.666, 1.944)	0.310 (−0.740, 1.371)	**2.670 (1.247, 4.113)**	1.385 (−0.290, 3.089)
	04	**1.020 (0.039, 2.011)**	1.024 (−0.022, 2.081)	0.699 (−0.672, 2.089)	0.639 (−0.471, 1.761)	**2.538 (1.032, 4.066)**	1.509 (−0.261, 3.311)
	05	**1.279 (0.249, 2.320)**	**1.339 (0.240, 2.449)**	0.858 (−0.580, 2.317)	0.921 (−0.243, 2.099)	**2.751 (1.170, 4.356)**	1.517 (−0.338, 3.408)
	06	**1.599 (0.526, 2.685)**	**1.642 (0.497, 2.801)**	1.254 (−0.244, 2.774)	1.139 (−0.075, 2.368)	**3.324 (1.677, 4.998)**	1.700 (−0.234, 3.671)
	07	**1.802 (0.688, 2.929)**	**1.894 (0.703, 3.098)**	1.439 (−0.115, 3.018)	**1.266 (0.009, 2.539)**	**3.631 (1.919, 5.371)**	1.654 (−0.353, 3.702)
	08	**1.954 (0.797, 3.125)**	**2.101 (0.864, 3.353)**	1.574 (−0.038, 3.213)	**1.379 (0.074, 2.701)**	**3.709 (1.930, 5.518)**	1.640 (−0.445, 3.769)
	09	**2.122 (0.921, 3.338)**	**2.327 (1.042, 3.628)**	**1.760 (0.088, 3.460)**	**1.488 (0.136, 2.859)**	**3.766 (1.921, 5.645)**	1.667 (−0.496, 3.878)
	010	**2.280 (1.033, 3.541)**	**2.476 (1.143, 3.827)**	**2.024 (0.290, 3.788)**	**1.675 (0.272, 3.097)**	**3.804 (1.892, 5.752)**	1.704 (−0.538, 3.997)
	011	**2.383 (1.092, 3.691)**	**2.601 (1.218, 4.002)**	**2.256 (0.458, 4.085)**	**1.691 (0.239, 3.165)**	**3.795 (1.816, 5.812)**	1.780 (−0.542, 4.156)
	012	**2.501 (1.165, 3.856)**	**2.699 (1.268, 4.151)**	**2.574 (0.712, 4.470)**	**1.682 (0.181, 3.207)**	**3.916 (1.870, 6.003)**	1.961 (−0.442, 4.422)
	013	**2.543 (1.160, 3.945)**	**2.742 (1.261, 4.244)**	**2.744 (0.818, 4.708)**	**1.590 (0.041, 3.164)**	**4.042 (1.927, 6.202)**	1.994 (−0.492, 4.542)
	014	**2.588 (1.161, 4.035)**	**2.773 (1.246, 4.324)**	**2.865 (0.876, 4.893)**	1.508 (−0.089, 3.129)	**4.173 (1.988, 6.404)**	2.240 (−0.329, 4.874)

Bold font indicates statistical significance.

CSD, circulatory system diseases; CEVD, Cerebrovascular Disease; CHD, Coronary Heart Disease; HF, Heart Failure.

**Figure 3:**
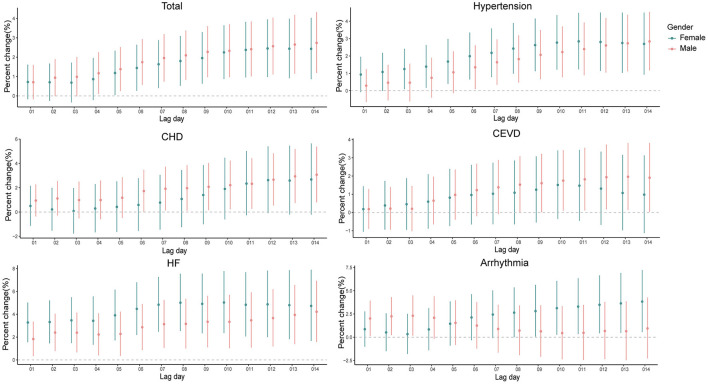
Percent changes and 95% confidence intervals (CIs) of daily hospital admissions for total and cause-specific circulatory system diseases by each 10 μg/m^3^ increase of PM_2.5_ concentrations stratified by gender at the cumulative-day lag models. HBP, hypertension; CHD, Coronary Heart Disease; CEVD, Cerebrovascular Disease; HF, Heart Failure.

The risk of hospital admissions seems to be higher in males for total CSD, CHD, and CEVD, and in females for hypertension, HF, and arrhythmia (Supplementary Figure 3 and Figure 3).

**Figure 4:**
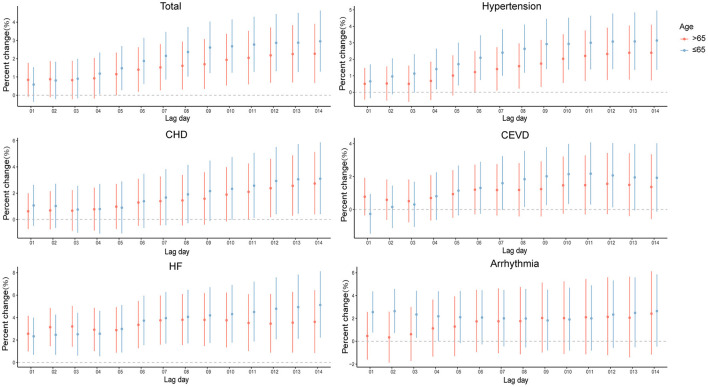
Percent changes and 95% confidence intervals (CIs) of daily hospital admissions for total and cause-specific circulatory system diseases by each 10 μg/m^3^ increase of PM_2.5_ concentrations stratified by age in the cumulative-day lag models. HBP, hypertension; CHD, Coronary Heart Disease; CEVD, Cerebrovascular Disease; HF, Heart Failure.

When analyses were stratified by age (≤65 and >65 years old), the results were not materially changed. In the elderly (>65 years old), significantly positive associations were observed of PM_2.5_ exposure with all the outcomes of interest in present study (Supplementary Figure 4 and Figure 4), slightly different from those in the younger (≤65 years old). As for the young people, PM_2.5_ levels were significantly associated with hospitalizations for CSD, except for arrhythmia.

**Figure 5:**
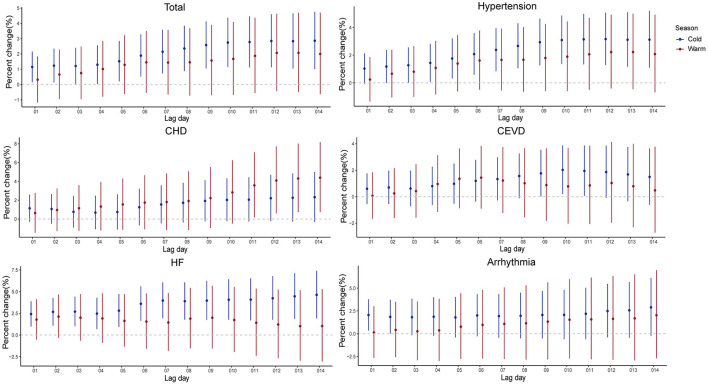
Percent changes and 95% confidence intervals (CIs) of daily hospital admissions for total and cause-specific circulatory system diseases by each 10 μg/m^3^ increase of PM_2.5_ concentrations stratified by season in the cumulative-day lag models. HBP, hypertension; CHD, Coronary Heart Disease; CEVD, Cerebrovascular Disease; HF, Heart Failure.

The impacts of PM_2.5_ on hospitalizations for CSD in cold seasons were stronger than those in warm seasons (Supplementary Figure 5 and Figure 5). In cold seasons, positive associations were observed in at least one exposure lag structure in present study, except for CHD. In warm seasons, hospitalizations for CHD and HF increased 2.618% (95% CI, 0.725–4.546%) and 2.769% (95% CI, 0.493–5.097%) per 10 μg/m^3^ elevation in PM_2.5_ levels, while there were no significant increases observed for total CSD, hypertension, CEVD, and arrhythmia.

The results of co-pollutant models were shown in Table 3. The associations remained largely unchanged when additionally adjusted for CO or/and O_3_, and were similar with additionally adjusted for all major air pollutants. Moreover, the results were similar when we changed the df for secular time (Supplementary Tables 1, 2 and Supplementary Figures 6–11) and adjusted the effect of COVID-19 (Supplementary Table 3), which illustrated the robustness of our findings.

**Table 3:** Percent changes and 95% CIs of hospital admissions associated with every 10 μg/m^3^ increase of PM_2.5_ under co-pollutant models.

**Model Type**	**^a^Total(PC, 95% CI, %)**	**^a^HBP (PC, 95% CI, %)**	**^a^CHD (PC, 95% CI, %)**	**^b^CVD (PC, 95% CI, %)**	**^a^HF (PC, 95% CI, %)**	**^a^ARR (PC, 95% CI, %)**
PM_2.5_	2.588 (1.161, 4.035)^*^	2.773 (1.246, 4.324)^*^	2.865 (0.876, 4.893)^*^	1.691 (0.239, 3.165)^*^	4.170 (1.988, 6.404)^*^	2.240 (−0.329, 4.874)
PM_2.5_ + CO	2.480 (0.997, 3.985)^*^	2.730 (1.132,4.354)^*^	2.781 (0.697,4.909)^*^	1.800 (0.279,3.345)^*^	3.867 (1.637,6.147)^*^	1.557 (−1.057,4.240)
PM_2.5_ + O_3_	2.626 (1.197, 4.075)^*^	2.804 (1.273,4.358)^*^	2.917 (0.927,4.946)^*^	1.664 (0.207,3.143)^*^	4.260 (2.068,6.498)^*^	2.425 (−0.149,5.066)
PM_2.5_ + CO + O_3_	2.494 (1.011, 3.999)^*^	2.739 (1.138,4.364)^*^	2.788 (0.705,4.914)^*^	1.782 (0.258,3.329)^*^	3.936 (1.702,6.219)^*^	1.658 (−0.966,4.352)
ALL	2.729 (1.227, 4.254)^*^	2.974 (1.344,4.629)^*^	3.055 (0.941,5.212)^*^	2.014 (0.455,3.596)^*^	3.979 (1.721,6.287)^*^	1.784 (−0.890,4.530)

PC, Percent change; HBP, Hypertension; CEVD, Cerebrovascular Disease; CHD, Coronary Heart Disease; HF, Heart Failure; ARR, arrhythmia.

a The peak lag effect of PM_2.5_ was lag014 in the cumulative lag models.

b The peak lag effect of PM_2.5_ was lag011 in the cumulative lag models.

* Means P < 0.05.

## 4. Discussion

In this study, we found that short-term exposure to PM_2.5_ was positively associated with hospitalizations for CSD, including total CSD, hypertension, CHD, CEVD, HF and arrhythmia, with significant lag effects. When analyses were stratified by age, gender, and season, there were no material changes of our findings, although the risk of hospitalizations seems to be higher among young people and cold seasons. Our results kept robust in the co-exposure models.

Though previous studies have reported relationships of PM_2.5_ with the risks of CSD, the results were largely inconsistent. Most studies found positive relationships of PM_2.5_ with increased risk of CSD. A multi-country time series study including 30 countries reported that every 10-μg/m^3^ elevation in PM_2.5_ concentrations was significantly relevant to a 0.12%, 0.42%, and 0.17% increase in cardiovascular diseases (CVD), acute myocardial infarction (AMI), and CHD on the same day (14). Furthermore, another study conducted in Beijing found that increasing PM_2.5_ levels were associated with hospitalizations for total CVD, CHD and atrial fibrillation (AF) (21). However, a few studies argued that there were no significantly associations of PM_2.5_ exposure with the risks of CSD (12, 15). Notably, the case data in above studies mainly came from government departments, which means that the data might be incomplete. Moreover, the confounding effect of time was not considered. Here, this time-series study based on more complete data were conducted and significantly positive associations of PM_2.5_ exposure with hospitalizations for CSD were observed, which was keeping with most studies.

The delayed effect of PM_2.5_ was also inconsistent in previous studies. Some studies across different countries showed that the impacts of PM_2.5_ on CSD mortality were peaked on the current day (22) and lag03 (13, 23, 24). A study in Kraków, Poland reported the delayed effects of PM_2.5_ levels on the risk of Myocardial Infarction (MI) admissions were observed at lag4 and lag6 (11). In our study, the earliest positive association between PM_2.5_ and hospitalizations for total CSD was at lag1 and peaked at lag6, as well as lag04 and lag014 in the cumulative-day lag model, which were longer than the hysteresis of other studies. The potential underlying reasons are as follows. First of all, the air quality of Ganzhou city is relatively good, and the average levels of PM_2.5_ from 2016 to 2020 was 37.38 μg/m^3^, which was significantly lower than that in other studies (21, 25). According to previous toxicological studies, PM_2.5_ exposure could lead to chronic systemic inflammation (26, 27), oxidative stress (28), stress hormone secretion (29–31) and vascular endothelial disfunction (32, 33), thereby causing to cardiovascular system damage. It takes several days from PM_2.5_ exposure to symptoms and hospitalization. Therefore, we speculated that exposure to higher levels of PM_2.5_ might have an acute effect on circulatory system health, while relatively low levels of PM_2.5_ tended to have delayed effects. Further studies are definitely needed to verify the hypothesis. Besides, in present study, hypertension and CHD accounted for relatively higher proportion of 47.0% and 14.2% in total CSD, respectively (Table 1). Worthy of note was that patients with hypertension and CHD are more inclined to self-medicate rather than to be hospitalized until their conditions worsen, which might be responsible for longer days' lag and underestimations of the impacts of PM_2.5_.

In this study, the cumulative-day lag model generally has higher estimates than the single-day lag model, with the greatest effects observed at lag 014. Similar results have been observed in other studies (34–36). The health effects of air pollutants usually last for several days, therefore, a cumulative lag model might be more accurate than a single-day lag model in assessing the health effects of air pollutants.

Our results regarding the positive associations of PM_2.5_ with CSD were correspondent with current mainstream understanding of the damage effects of PM_2.5_ on the circulatory system. Previous toxicological studies (28) have shown that PM_2.5_ is inhaled into lung through respiration, causing lung inflammation. The particles and inflammatory mediators released by alveolar macrophages could also enter the blood circulation system directly through the capillaries in the lungs and cause systemic inflammation and vascular endothelial dysfunction (37). These responses may underlie PM-induced circulatory system damage. PM_2.5_ could also activate the hypothalamic–pituitary–adrenal (HPA) axis, triggering an increase of stress hormones release, thereby causing vasoconstriction, increased blood pressure and a series of pathological reactions (29). However, full details regarding the biological mechanisms remain largely unclear and warrant further study.

The associations between PM_2.5_ and hospitalizations for CSD were not materially changed in different gender subgroups. The impacts of PM_2.5_ on the hospitalizations for arrhythmia seemed to be stronger in females compared with males, consistent with some studies (35–37), which might be attributable to more vulnerable biological systems of females.

In age subgroups, we found that younger people (≤65 years old) showed greater sensitivity to PM_2.5_ exposure in total CSD, hypertension, CHD, CEVD, and heart failure. In addition to air pollution, numerous factors such as occupational exposure (38), lifestyles (39), and even social status (40) and psychological factors (8) could also affect the health of circulatory system. Compared with the elderly, younger people tend to spend more time outdoors and are more vulnerable to harmful ambient hazards and occupational factors, such as industrial dust, chemicals, and noise (41), which can explain why younger people are more susceptible to PM_2.5_. Overall, more researches are warranted to probe the potential modifiers in age and sex on associations of PM_2.5_ exposure with the risks of CSD.

In our study, the impacts of PM_2.5_ on the CSD, except for CHD, dominated during the cold seasons. The differences in PM_2.5_ levels and compositions might be accounted for the seasonal variations in the relationships of PM_2.5_ exposure with hospitalizations for CSD. The mean levels of PM_2.5_ during cold seasons from 2016 to 2020 in Ganzhou is 43.62 μg/m^3^, significantly higher than that in warm seasons (31.22 μg/m^3^), shown in Supplementary Table 2. Furthermore, some constituents in PM_2.5_, including polycyclic aromatic hydrocarbons (PAHs) and metals, which were acknowledged to cause damage to the circulatory system (42, 43), increased significantly compared with warm seasons according to related studies (44, 45).

Profound elucidation for the exposure-response relationship is essential for public health policy formulation regarding the limit for PM_2.5_. In this study, the exposure-response curves were approximately linear with relatively steeper increases at higher concentrations of PM2.5 (>110 mg/m^3^ for HF, >100 mg/m^3^ for other circulatory system diseases). A series of previous studies also reported similar linear exposure-response relationships (21, 36, 46–50). For example, a study in Beijing found that the hospitalizations for ischemic stroke had a stable increase at lower concentrations (<100 μg/m^3^) and a steeper increment at higher concentrations of PM_2.5_ (46).

Notably, the average 24h concentration of PM_2.5_ in Ganzhou from 2016 to 2020 was 37 μg/m^3^, lower than current National Ambient Air Quality Standard (NAAQS) for PM_2.5_ (75 μg/m^3^) (51). However, significantly positive associations of PM_2.5_ and hospitalizations for CSD were still observed. Our findings were consistent with some studies. In a study of 200 Chinese cities (52), which included 58.52 million hospital admissions, the positive relationships of PM_2.5_ with hospitalizations were observed when the daily levels met the current NAAQS (75 μg/m^3^). Furthermore, a recent analysis of Europe (53) also revealed that long-term low levels of PM_2.5_ exposure was related to the morbidity of stroke and CHD. Additionally, a study in USA (54) also reported the deleterious effects of PM_2.5_ at levels below the specified limits. From the perspective of public health, our study suggests that more stringent PM_2.5_ standard limits than current NAAQS should be established to minimize the harmful effects of ambient PM_2.5_.

In the co-pollutant models, after adjusting CO or/and O_3_, and other major pollutants, the hospitalizations for CSD per 10-μg/m^3^ elevation of PM_2.5_ still significantly increased, indicating that the impacts of PM_2.5_ on the risks of CSD were robust, in keeping with most studies (21, 23, 55).

There are several strengths in the current study. Firstly, this study estimated the associations between PM_2.5_ levels and the risk of hospitalizations for CSD in Ganzhou for the first time. Besides, hospital admission data was selected as the effect indicator, which was more sensitive than mortality and has great public health implications.

However, several limitations should also be considered. First, using outdoor air pollution measured at outdoor fixed sited monitors as a proxy for individual exposure levels might lead to the misestimation of the exposure assessment. Secondly, our case data were only collected from one hospital in Ganzhou, which was inevitably to the ecological fallacy and the extrapolation of our research results were limited, to a certain extent. In addition, confounding factors such as smoking, alcohol consumption, occupation and education levels were not considered in the analysis due to lack of information. Finally, we were unable to evaluate the long-term influence of PM_2.5_ on the CSD under the time-series analysis design. Therefore, more well-designed studies are needed to explore the short-term and long-term impacts of PM_2.5_ exposure on the incidence of CSD in depth.

## 5. Conclusion

In this study, we found significantly positive associations of relatively low PM_2.5_ exposure with daily hospitalizations for total and cause-specific CSD in Ganzhou. And the associations varied in age, gender, and season subgroups. Our findings provide substantial insight regarding the effects of PM_2.5_ exposure on CSD, which may provide evidence of stricter limits on PM_2.5_ concentrations and help local policymakers to formulate or promulgate prevention policies.

## Data availability statement

The raw data supporting the conclusions of this article will be made available by the authors, without undue reservation.

## Author contributions

XY contributed to conception, the design of the study, data analysis, and wrote the first draft of the manuscript. XC contributed to data analysis and the design of the study. YG, DW, WQ, CZ, and MZ contributed to manuscript revision. WC and XZ contributed to conception, the design of the study, funding acquisition, and project administration. All authors contributed to manuscript revision, read, and approved the submitted version.
