# Supplementary material for: Associations between short-term PM2.5 exposure and daily hospital admissions for circulatory system diseases in Ganzhou, China: A time series study
Source: Front Public Health. 2023 Mar 9;11:1134516. doi: 10.3389/fpubh.2023.1134516 (PMC10034184; doi:10.3389/fpubh.2023.1134516)
Supplement: Supplementary file 1 [file Table_1.DOCX]

Supplementary Material

**Associations between** **short-term PM_2.5_ exposure and daily hospital admissions for circulatory system diseases in Ganzhou, China: a time series study**

Xiaojie You^1, 2^, Xiuyu Cao^1, 2^, You Guo^3, 4, 5^, Dongming Wang^1, 2^, Weihong Qiu^1, 2^, Chuanfei Zhou^3, 4, 5^, Min Zhou^1, 2^, Weihong Chen^1, 2, *^, Xiaokang Zhang^3, 4, 5, *^

*** Correspondence:**

Corresponding Author:

Weihong Chen: [wchen@mails.tjmu.edu.cn](mailto:wchen@mails.tjmu.edu.cn)

Xiaokang Zhang: [zhangxiaokaju@163.com](mailto:zhangxiaokaju@163.com)

# Supplementary Figures and Tables

## Supplementary Tables

| **Supplementary Table 1 Percent changes and 95% CIs of hospital admissions for circulatory system diseases by each 10 μg/m^3^ increase of PM_2.5_ concentrations at different lag structures in the single-pollutant model (dfs for secular time is 7 per year)** | | | | | | | |
| --- | --- | --- | --- | --- | --- | --- | --- |
| **Lag Type** | **Lag day** | **Total CSD** | **Hypertension** | **CHD** | **CEVD** | **HF** | **Arrhythmia** |
| single-lag | 0 | 0.1 (-0.642,0.848) | -0.016 (-0.816,0.79) | 0.291 (-0.745,1.338) | -0.013 (-0.851,0.832) | **1.393 (0.281,2.518)** | 0.727 (-0.621,2.094) |
|  | 1 | **0.779 (0.053,1.509)** | 0.655 (-0.126,1.442) | 0.74 (-0.284,1.775) | 0.592 (-0.219,1.41) | **2.252 (1.147,3.368)** | **1.592 (0.278,2.924)** |
|  | 2 | 0.466 (-0.243,1.179) | 0.49 (-0.272,1.257) | 0.248 (-0.753,1.26) | 0.444 (-0.348,1.242) | **1.628 (0.544,2.723)** | 0.527 (-0.762,1.832) |
|  | 3 | 0.205 (-0.496,0.911) | 0.336 (-0.419,1.097) | -0.192 (-1.183,0.809) | 0.294 (-0.491,1.086) | 0.864 (-0.202,1.941) | 0.327 (-0.948,1.618) |
|  | 4 | 0.592 (-0.099,1.288) | 0.72 (-0.026,1.471) | 0.271 (-0.709,1.261) | **0.989 (0.207,1.776)** | 0.416 (-0.632,1.476) | 0.533 (-0.728,1.81) |
|  | 5 | **0.843 (0.158,1.533)** | **1.041 (0.301,1.786)** | 0.565 (-0.408,1.548) | **0.962 (0.184,1.745)** | **1.055 (0.015,2.106)** | 0.368 (-0.885,1.636) |
|  | 6 | **1.011 (0.332,1.695)** | **1.036 (0.303,1.774)** | 1.096 (0.131,2.069) | **0.87 (0.098,1.648)** | **1.901 (0.869,2.944)** | 0.75 (-0.497,2.013) |
|  | 7 | **0.821 (0.142,1.505)** | **0.99 (0.256,1.728)** | 0.708 (-0.257,1.682) | **0.794 (0.022,1.572)** | **1.301 (0.268,2.345)** | 0.182 (-1.063,1.444) |
|  | 8 | 0.625 (-0.057,1.311) | **0.833 (0.097,1.576)** | 0.505 (-0.463,1.482) | 0.663 (-0.112,1.445) | 0.707 (-0.324,1.749) | 0.097 (-1.149,1.359) |
|  | 9 | 0.658 (-0.019,1.34) | **0.879 (0.148,1.615)** | 0.644 (-0.316,1.614) | 0.661 (-0.111,1.439) | 0.52 (-0.503,1.553) | 0.237 (-0.998,1.489) |
|  | 10 | 0.601 (-0.08,1.285) | 0.593 (-0.142,1.333) | **0.97 (0.004,1.945)** | **0.87 (0.092,1.654)** | 0.381 (-0.641,1.414) | 0.238 (-1.001,1.492) |
|  | 11 | 0.402 (-0.281,1.09) | 0.49 (-0.249,1.234) | 0.871 (-0.102,1.854) | 0.286 (-0.494,1.072) | 0.192 (-0.834,1.228) | 0.337 (-0.909,1.599) |
|  | 12 | 0.427 (-0.261,1.119) | 0.388 (-0.355,1.137) | 1.19 (0.211,2.179) | 0.148 (-0.634,0.937) | 0.503 (-0.527,1.544) | 0.704 (-0.55,1.973) |
|  | 13 | 0.14 (-0.548,0.833) | 0.156 (-0.589,0.906) | 0.627 (-0.354,1.618) | -0.122 (-0.906,0.667) | 0.506 (-0.528,1.55) | 0.173 (-1.083,1.445) |
|  | 14 | 0.138 (-0.55,0.831) | 0.109 (-0.636,0.859) | 0.375 (-0.604,1.363) | -0.116 (-0.9,0.675) | 0.493 (-0.543,1.539) | 0.95 (-0.313,2.229) |
|  |  |  |  |  |  |  |  |
| Cumulative-lag | 1 | 0.547 (-0.265,1.365) | 0.4 (-0.473,1.28) | 0.171 (-0.749,1.101) | 0.629 (-0.509,1.78) | **2.267 (1.038,3.511)** | **1.45 (0.001,2.919)** |
|  | 2 | 0.655 (-0.22,1.537) | 0.55 (-0.389,1.498) | 0.259 (-0.728,1.256) | 0.616 (-0.609,1.857) | **2.589 (1.257,3.939)** | 1.378 (-0.182,2.962) |
|  | 3 | 0.65 (-0.282,1.592) | 0.611 (-0.391,1.622) | 0.275 (-0.775,1.336) | 0.455 (-0.85,1.778) | **2.621 (1.197,4.066)** | 1.317 (-0.344,3.005) |
|  | 4 | 0.809 (-0.179,1.808) | 0.814 (-0.247,1.888) | 0.618 (-0.489,1.737) | 0.493 (-0.89,1.895) | **2.468 (0.959,3.999)** | 1.423 (-0.331,3.208) |
|  | 5 | **1.049 (0.011,2.099)** | 1.115 (-0.002,2.244) | 0.909 (-0.249,2.08) | 0.626 (-0.827,2.1) | **2.673 (1.088,4.282)** | 1.429 (-0.41,3.301) |
|  | 6 | **1.361 (0.278,2.456)** | **1.409 (0.243,2.589)** | 1.133 (-0.072,2.352) | 1.021 (-0.492,2.557) | **3.255 (1.603,4.934)** | 1.6 (-0.315,3.552) |
|  | 7 | **1.548 (0.422,2.686)** | **1.648 (0.436,2.876)** | **1.253 (0.006,2.516)** | 1.192 (-0.379,2.788) | **3.566 (1.849,5.313)** | 1.543 (-0.448,3.573) |
|  | 8 | **1.687 (0.518,2.87)** | **1.849 (0.588,3.125)** | **1.364 (0.07,2.675)** | 1.316 (-0.315,2.973) | **3.632 (1.847,5.449)** | 1.499 (-0.569,3.611) |
|  | 9 | **1.84 (0.626,3.069)** | **2.062 (0.752,3.39)** | **1.468 (0.127,2.827)** | 1.493 (-0.198,3.213) | **3.684 (1.83,5.571)** | 1.508 (-0.639,3.701) |
|  | 10 | **1.982 (0.722,3.259)** | **2.197 (0.837,3.576)** | **1.651 (0.263,3.059)** | 1.745 (-0.01,3.53) | **3.717 (1.795,5.675)** | 1.526 (-0.7,3.801) |
|  | 11 | **2.068 (0.761,3.392)** | **2.307 (0.896,3.737)** | **1.66 (0.222,3.117)** | **1.958 (0.139,3.811)** | **3.7 (1.71,5.73)** | 1.577 (-0.726,3.934) |
|  | 12 | **2.174 (0.822,3.545)** | **2.391 (0.929,3.873)** | **1.644 (0.16,3.151)** | **2.264 (0.379,4.185)** | **3.83 (1.771,5.931)** | 1.737 (-0.646,4.176) |
|  | 13 | **2.192 (0.792,3.612)** | **2.41 (0.897,3.945)** | **1.536 (0.002,3.093)** | **2.412 (0.459,4.403)** | **3.957 (1.826,6.132)** | 1.746 (-0.718,4.271) |
|  | 14 | **2.21 (0.763,3.678)** | **2.413 (0.85,4.001)** | 1.437 (-0.145,3.045) | **2.518 (0.501,4.574)** | **4.085 (1.882,6.335)** | 2.008 (-0.536,4.618) |
| Bold font indicates statistical significance. Abbreviations: CSD, circulatory system diseases; CEVD, Cerebrovascular Disease; CHD, Coronary Heart Disease; HF, Heart Failure. | | | | | | | |

| **Supplementary Table 2 Percent changes and 95% CIs of hospital admissions for total CSD by each 10 μg/m^3^ increase of PM_2.5_ concentrations in cold and warm seasons at different lag structures（df for secular time is 3 per year）** | | | |
| --- | --- | --- | --- |
| **Lag Type** | **Lag day** | **Total CSD (Cold)** | **Total CSD (Warm)** |
| single-lag | 0 | 0.128 (-0.781,1.046) | -0.002 (-1.367,1.382) |
|  | 1 | 0.9 (-0.004,1.812) | 0.635 (-0.676,1.964) |
|  | 2 | 0.276 (-0.601,1.161) | 0.93 (-0.367,2.243) |
|  | 3 | -0.014 (-0.871,0.85) | 0.495 (-0.805,1.813) |
|  | 4 | 0.226 (-0.609,1.068) | 0.851 (-0.45,2.17) |
|  | 5 | 0.603 (-0.215,1.428) | 0.986 (-0.328,2.318) |
|  | 6 | **0.931 (0.127,1.742)** | 0.674 (-0.64,2.005) |
|  | 7 | **0.822 (0.016,1.633)** | 0.159 (-1.146,1.482) |
|  | 8 | 0.65 (-0.158,1.464) | 0.265 (-1.043,1.59) |
|  | 9 | 0.664 (-0.136,1.469) | 0.49 (-0.828,1.825) |
|  | 10 | 0.475 (-0.327,1.283) | 0.581 (-0.743,1.923) |
|  | 11 | 0.083 (-0.725,0.897) | 0.614 (-0.699,1.945) |
|  | 12 | 0.08 (-0.734,0.9) | 0.666 (-0.644,1.994) |
|  | 13 | -0.171 (-0.99,0.654) | -0.111 (-1.415,1.211) |
|  | 14 | -0.059 (-0.873,0.761) | -0.284 (-1.581,1.03) |
|  |  |  |  |
| Cumulative-lag | 1 | 0.641 (-0.373,1.665) | 0.414 (-1.059,1.909) |
|  | 2 | 0.649 (-0.457,1.767) | 0.756 (-0.823,2.36) |
|  | 3 | 0.562 (-0.627,1.764) | 0.859 (-0.826,2.571) |
|  | 4 | 0.586 (-0.68,1.868) | 1.115 (-0.668,2.929) |
|  | 5 | 0.776 (-0.554,2.124) | 1.38 (-0.498,3.293) |
|  | 6 | 1.149 (-0.236,2.553) | 1.539 (-0.432,3.549) |
|  | 7 | 1.379 (-0.057,2.835) | 1.535 (-0.524,3.636) |
|  | 8 | **1.582 (0.089,3.097)** | 1.568 (-0.577,3.759) |
|  | 9 | **1.796 (0.244,3.372)** | 1.685 (-0.543,3.964) |
|  | 10 | **1.964 (0.35,3.604)** | 1.803 (-0.515,4.175) |
|  | 11 | **2.014 (0.336,3.72)** | 1.993 (-0.399,4.441) |
|  | 12 | **2.091 (0.348,3.864)** | 2.18 (-0.28,4.7) |
|  | 13 | **2.019 (0.212,3.859)** | 2.184 (-0.339,4.771) |
|  | 14 | **1.929 (0.059,3.835)** | 2.118 (-0.468,4.772) |

| **Supplementary Table 3 Percent changes and 95% CIs of hospital admissions for circulatory system diseases by each 10 μg/m^3^ increase of PM_2.5_ concentrations at different lag structures after adjusting the COVID-19 in the single-pollutant model** | | | | | | | |
| --- | --- | --- | --- | --- | --- | --- | --- |
| **Lag Type** | **Lag day** | **Total CSD** | **Hypertension** | **CHD** | **CEVD** | **HF** | **Arrhythmia** |
| Single-lag | 0 | -0.132 (-0.968,0.712) | -0.275 (-1.196,0.654) | -0.114 (-1.453,1.243) | -0.573 (-1.588,0.451) | 0.772 (-0.549,2.11) | 0.99 (-0.509,2.512) |
|  | 1 | **0.97 (0.121,1.826)** | 0.935 (-0.004,1.883) | 0.508 (-0.869,1.904) | 0.677 (-0.36,1.724) | 1.267 (-0.005,2.556) | **1.887 (0.405,3.39)** |
|  | 2 | 0.233 (-0.592,1.063) | 0.322 (-0.59,1.243) | -0.456 (-1.789,0.895) | 0.281 (-0.732,1.305) | 0.49 (-0.829,1.827) | 0.462 (-0.984,1.929) |
|  | 3 | -0.143 (-0.939,0.659) | 0.132 (-0.75,1.022) | -1.334 (-2.621, -0.03) | -0.136 (-1.116,0.853) | -0.255 (-1.521,1.028) | 0.488 (-0.941,1.938) |
|  | 4 | 0.257 (-0.517,1.037) | 0.525 (-0.333,1.39) | -0.521 (-1.782,0.757) | 0.682 (-0.27,1.644) | -0.818 (-2.045,0.424) | 0.318 (-1.1,1.756) |
|  | 5 | 0.663 (-0.099,1.43) | **1.032 (0.189,1.881)** | 0.036 (-1.207,1.295) | 0.531 (-0.404,1.475) | 0.613 (-0.619,1.859) | -0.17 (-1.572,1.253) |
|  | 6 | **1.108 (0.357,1.866)** | **1.086 (0.254,1.925)** | **1.346 (0.106,2.6)** | 0.747 (-0.176,1.678) | **1.969 (0.745,3.208)** | 0.524 (-0.868,1.935) |
|  | 7 | **0.89 (0.135,1.651)** | **1.072 (0.237,1.915)** | 0.669 (-0.567,1.92) | 0.862 (-0.062,1.794) | 0.926 (-0.29,2.156) | -0.204 (-1.598,1.209) |
|  | 8 | 0.535 (-0.225,1.301) | 0.804 (-0.039,1.653) | 0.15 (-1.085,1.4) | 0.86 (-0.067,1.796) | -0.448 (-1.598,0.715) | -0.157 (-1.55,1.256) |
|  | 9 | 0.441 (-0.309,1.197) | 0.622 (-0.207,1.459) | 0.05 (-1.17,1.285) | 0.791 (-0.126,1.716) | -0.358 (-1.493,0.792) | -0.123 (-1.499,1.273) |
|  | 10 | 0.176 (-0.576,0.933) | 0.036 (-0.793,0.873) | 0.017 (-1.207,1.256) | **1.045 (0.231,1.87)** | -0.274 (-1.405,0.871) | -0.396 (-1.769,0.996) |
|  | 11 | -0.367 (-1.119,0.391) | -0.355 (-1.186,0.482) | -0.455 (-1.683,0.789) | -0.315 (-1.232,0.611) | -0.571 (-1.704,0.575) | -0.237 (-1.623,1.169) |
|  | 12 | -0.326 (-1.086,0.439) | -0.474 (-1.311,0.37) | 0.406 (-0.829,1.656) | -0.668 (-1.588,0.26) | -0.006 (-1.153,1.154) | 0.282 (-1.111,1.694) |
|  | 13 | -0.631 (-1.395,0.138) | -0.807 (-1.648,0.041) | -0.051 (-1.292,1.206) | -0.777 (-1.701,0.155) | 0.042 (-1.113,1.21) | -0.33 (-1.728,1.088) |
|  | 14 | -0.52 (-1.283,0.248) | -0.605 (-1.449,0.247) | -0.422 (-1.661,0.833) | -0.649 (-1.577,0.288) | -0.173 (-1.328,0.996) | 0.499 (-0.904,1.921) |
|  |  |  |  |  |  |  |  |
| Cumulative-lag | 1 | 0.544 (-0.425,1.521) | 0.446 (-0.62,1.524) | 1.442 (-0.089,2.996) | 0.114 (-1.046,1.288) | **2.375 (0.977,3.793)** | **1.741 (0.096,3.413)** |
|  | 2 | 0.614 (-0.485,1.725) | 0.61 (-0.602,1.836) | 1.368 (-0.194,2.956) | 0.301 (-1.011,1.63) | **2.705 (1.193,4.24)** | 1.589 (-0.179,3.388) |
|  | 3 | 0.502 (-0.717,1.736) | 0.642 (-0.702,2.005) | 1.184 (-0.505,2.902) | 0.255 (-1.193,1.724) | **2.772 (1.158,4.411)** | 1.562 (-0.319,3.48) |
|  | 4 | 0.637 (-0.705,1.996) | 0.951 (-0.527,2.451) | 0.542 (-1.282,2.4) | 0.692 (-0.895,2.306) | **2.492 (0.785,4.227)** | 1.571 (-0.421,3.602) |
|  | 5 | 1.025 (-0.432,2.504) | 1.582 (-0.022,3.212) | 0.741 (-1.184,2.703) | 1.053 (-0.664,2.799) | **2.717 (0.922,4.544)** | 1.339 (-0.751,3.474) |
|  | 6 | **1.768 (0.209,3.352)** | **2.283 (0.561,4.034)** | 1.614 (-0.379,3.647) | 1.578 (-0.26,3.449) | **3.392 (1.523,5.295)** | 1.438 (-0.738,3.662) |
|  | 7 | **2.302 (0.626,4.005)** | **2.927 (1.072,4.815)** | 1.949 (-0.138,4.079) | **2.02 (0.046,4.033)** | **3.695 (1.749,5.677)** | 1.26 (-1,3.572) |
|  | 8 | **2.717 (0.905,4.561)** | **3.553 (1.541,5.606)** | 1.629 (-0.562,3.868) | **2.648 (0.51,4.832)** | **3.523 (1.497,5.59)** | 1.194 (-1.156,3.6) |
|  | 9 | **3.134 (1.17,5.136)** | **4.129 (1.934,6.372)** | 1.386 (-0.925,3.751) | **3.305 (0.979,5.684)** | **3.414 (1.305,5.568)** | 1.127 (-1.318,3.633) |
|  | 10 | **3.419 (1.296,5.587)** | **4.355 (1.975,6.79)** | 2.166 (-0.948,5.378) | **4.198 (1.662,6.797)** | **3.327 (1.131,5.571)** | 0.986 (-1.557,3.595) |
|  | 11 | **3.349 (1.054,5.695)** | **4.369 (1.792,7.01)** | 1.734 (-1.535,5.112) | **4.215 (1.48,7.025)** | **3.134 (0.851,5.468)** | 0.906 (-1.737,3.621) |
|  | 12 | **3.183 (0.713,5.713)** | **3.886 (1.135,6.713)** | 1.062 (-1.578,3.772) | **4.702 (1.671,7.824)** | **3.154 (0.787,5.578)** | 0.991 (-1.754,3.812) |
|  | 13 | **2.989 (0.294,5.757)** | **3.638 (0.639,6.727)** | 1.093 (-1.679,3.944) | **4.301 (1.017,7.692)** | **3.19 (0.737,5.703)** | 0.882 (-1.962,3.809) |
|  | 14 | **2.947 (0.111,5.783)** | **3.425 (0.127,6.743)** | 1.109 (-1.939,4.187) | **4.235 (0.768,7.692)** | **3.217 (0.496,5.938)** | 0.769 (-2.216,3.754) |

| **Supplementary** **Table 4 The characteristics of ambient air pollutants in cold and warm seasons of Ganzhou, 2016-2020** | | | | | | | |
| --- | --- | --- | --- | --- | --- | --- | --- |
| Air pollutants | Season | Mean | Min | P25 | Median | P75 | Max |
| PM_2.5_ (μg/m³) | Cold | 43.62 | 6.00 | 25.00 | 39.00 | 57.00 | 184.00 |
|  | Warm | 31.22 | 7.00 | 21.00 | 29.00 | 39.00 | 87.00 |
| PM_10_ (μg/m³) | Cold | 69.52 | 11.00 | 39.00 | 611.00 | 91.00 | 246.00 |
|  | Warm | 50.73 | 12.00 | 35.00 | 47.00 | 63.00 | 131.00 |
| SO_2_ (μg/m³) | Cold | 19.63 | 2.00 | 10.00 | 16.00 | 26.50 | 73.00 |
|  | Warm | 17.85 | 3.00 | 11.00 | 16.00 | 22.00 | 63.00 |
| NO_2_ (μg/m³) | Cold | 28.39 | 4.00 | 18.00 | 25.00 | 35.00 | 84.00 |
|  | Warm | 17.20 | 5.00 | 12.00 | 15.00 | 20.00 | 57.00 |
| CO (mg/m³) | Cold | 1.36 | 0.60 | 1.10 | 1.32 | 1.58 | 2.90 |
|  | Warm | 1.13 | 0.60 | 0.93 | 1.10 | 1.32 | 2.13 |
| O_3_ (μg/m³) | Cold | 77.53 | 7.00 | 48.00 | 74.00 | 104.00 | 224.00 |
|  | Warm | 103.30 | 27.00 | 76.00 | 100.00 | 129.20 | 213.00 |

## Supplementary Figures


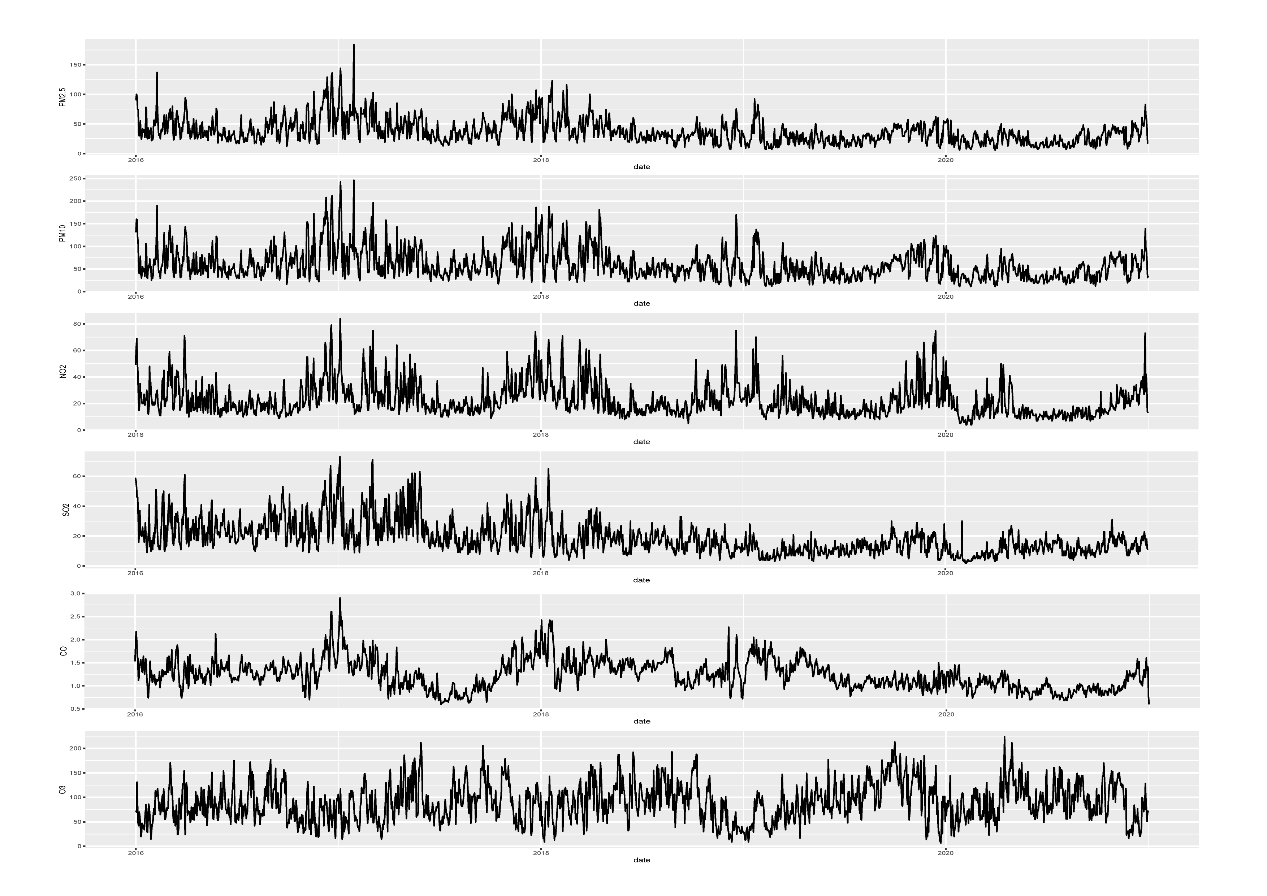


**Supplementary Figure 1.** **Temporal patterns of the air pollutants in Ganzhou, China during 2016-2020**


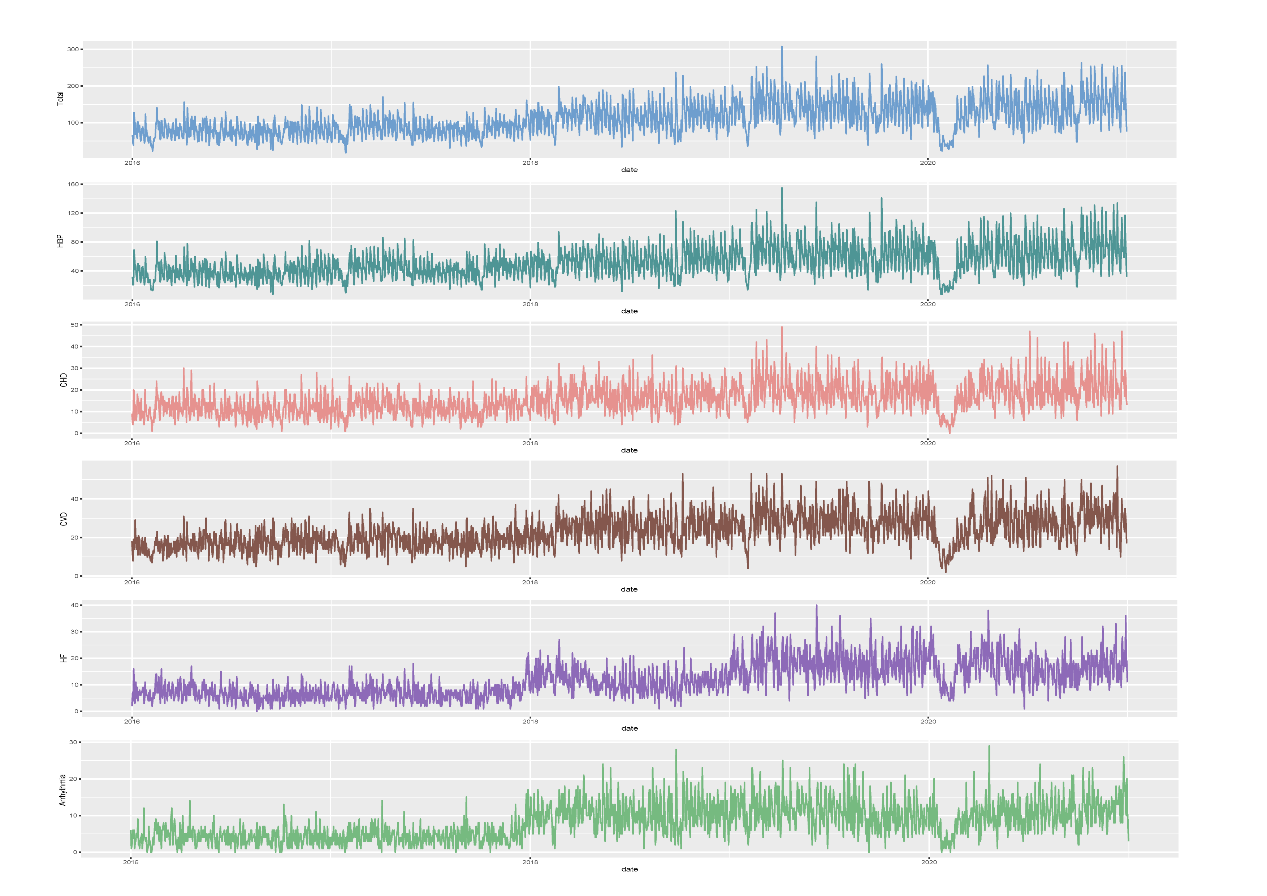


**Supplementary Figure 2. Temporal patterns of hospital admissions for circulatory system diseases in Ganzhou, China during 2016-2020**


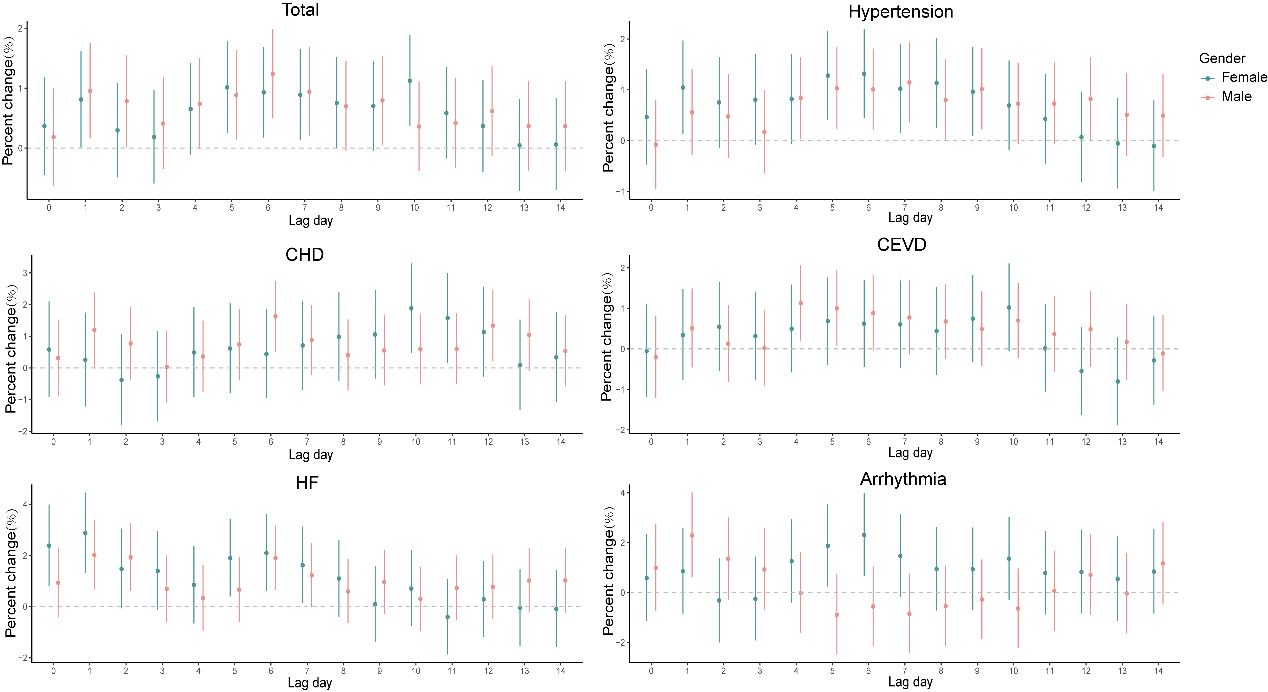


**Supplementary Figure 3. Percent changes and 95% confidence intervals (CIs) of daily hospital admissions for total and cause-specific circulatory system diseases by each 10 μg/m^3^ increase of PM_2.5_ concentrations stratified by gender in the single-day lag models.**

Abbreviations: HBP, hypertension; CHD, Coronary Heart Disease; CEVD, Cerebrovascular Disease; HF, Heart Failure.


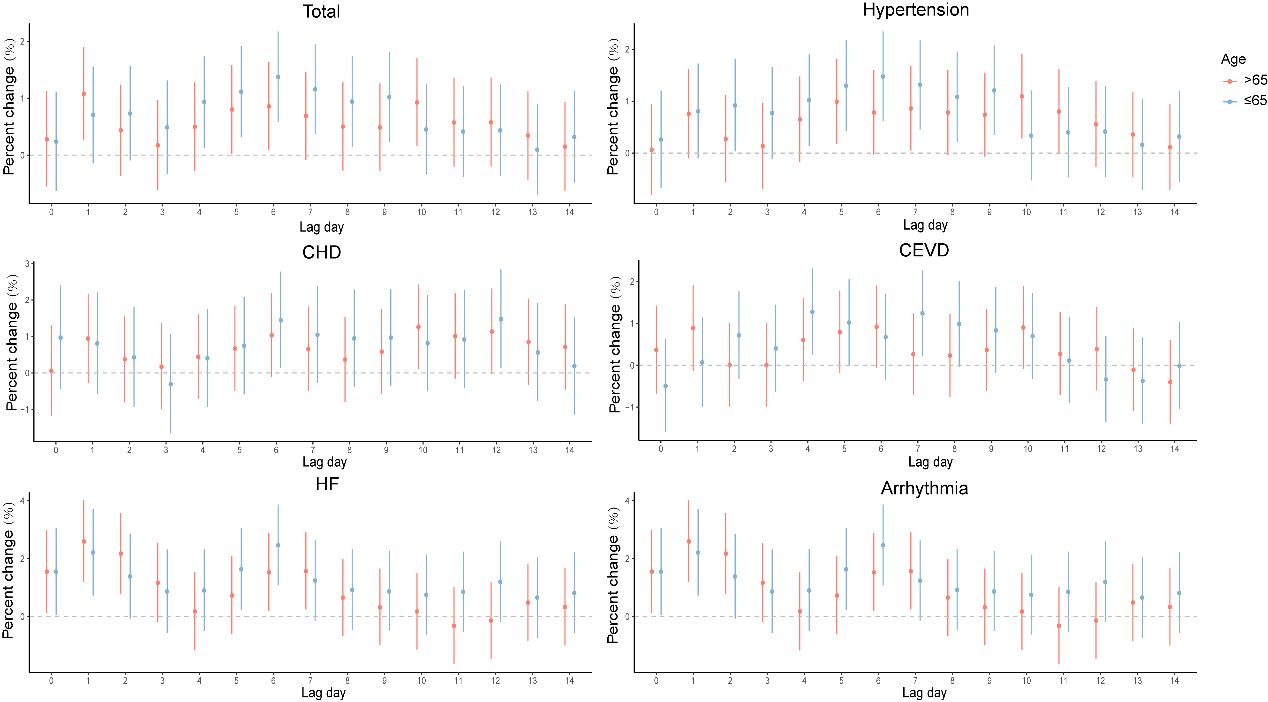


**Supplementary Figure 4. Percent changes and 95% confidence intervals (CIs) of daily hospital admissions for total and cause-specific circulatory system diseases by each 10 μg/m^3^ increase of PM_2.5_ concentrations stratified by age in the single-day lag models.**

Abbreviations: HBP, hypertension; CHD, Coronary Heart Disease; CEVD, Cerebrovascular Disease; HF, Heart Failure.


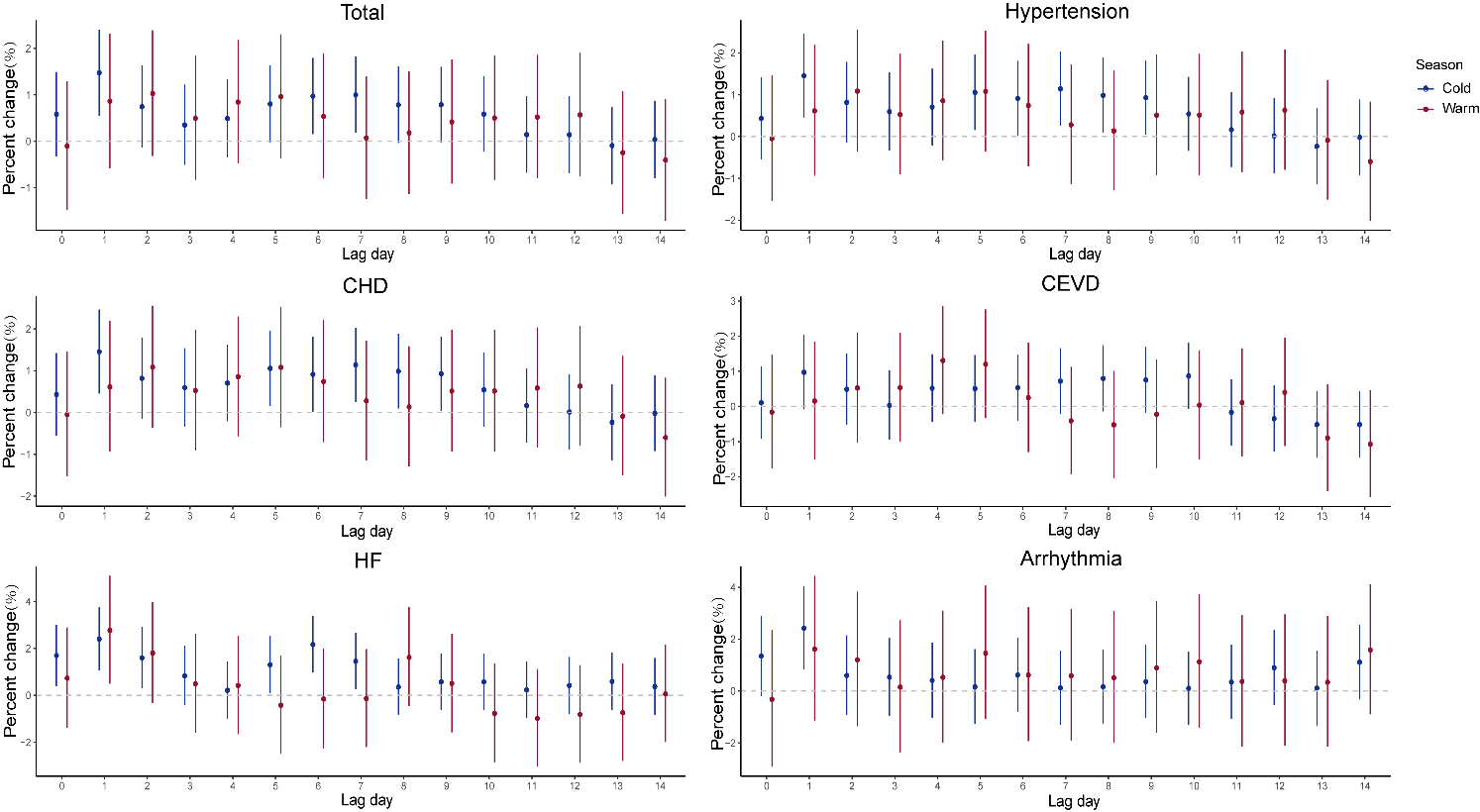


**Supplementary Figure 5. Percent changes and 95% confidence intervals (CIs) of daily hospital admissions for total and cause-specific circulatory diseases by each 10 μg/m^3^ increase of PM_2.5_ concentrations stratified by season in the single-day lag models.**

Abbreviations: HBP, hypertension; CHD, Coronary Heart Disease; CEVD, Cerebrovascular Disease; HF, Heart Failure.

**
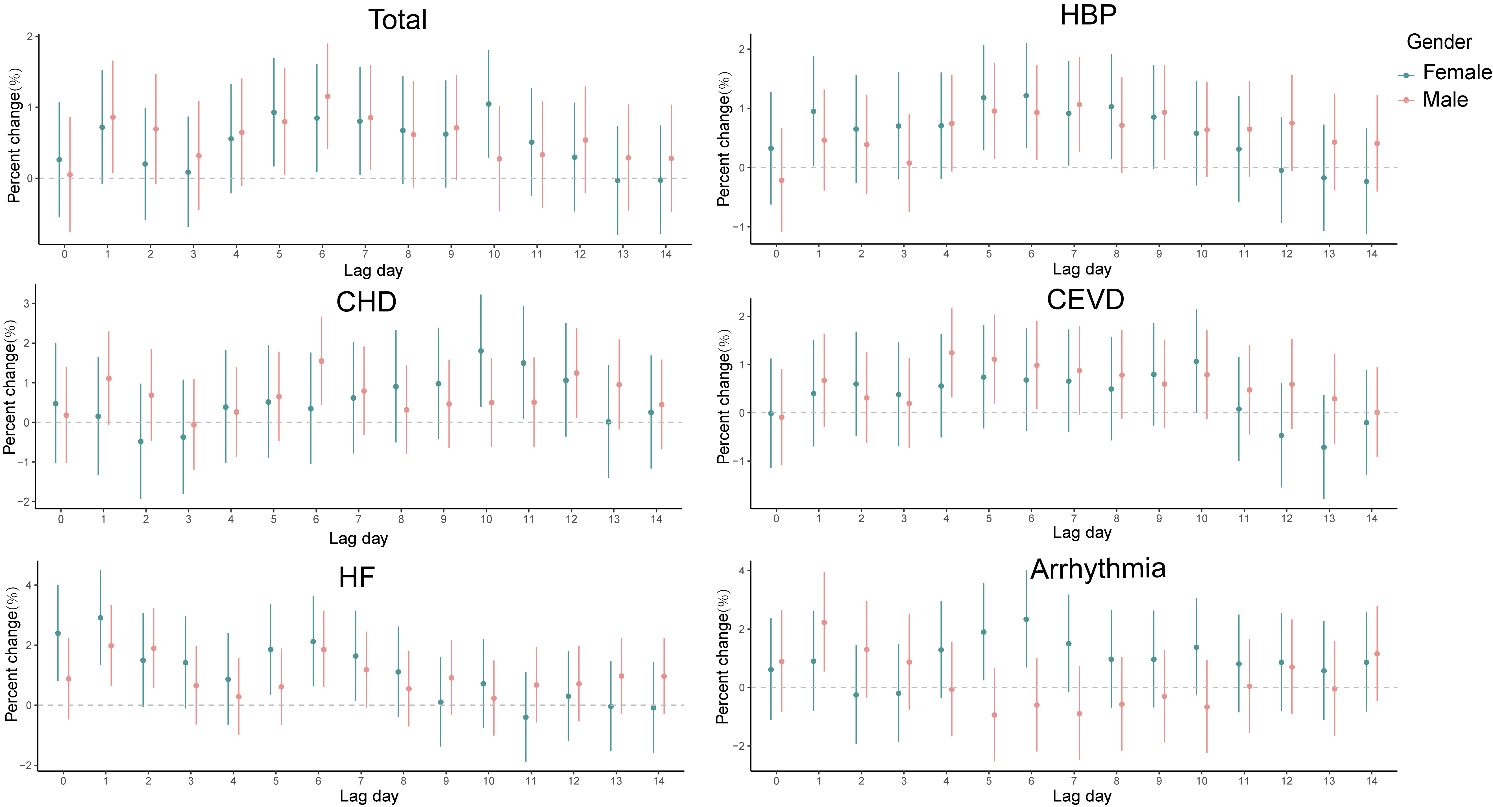
**

**Supplementary Figure 6. Percent changes and 95% CIs of daily hospital admissions for total and cause-specific circulatory system diseases by each 10 μg/m^3^ increase of PM_2.5_ concentrations stratified by gender in the single-day lag models (dfs for secular time is 7 per year).** Abbreviations: HBP, hypertension; CHD, Coronary Heart Disease; CEVD, Cerebrovascular Disease; HF, Heart Failure.


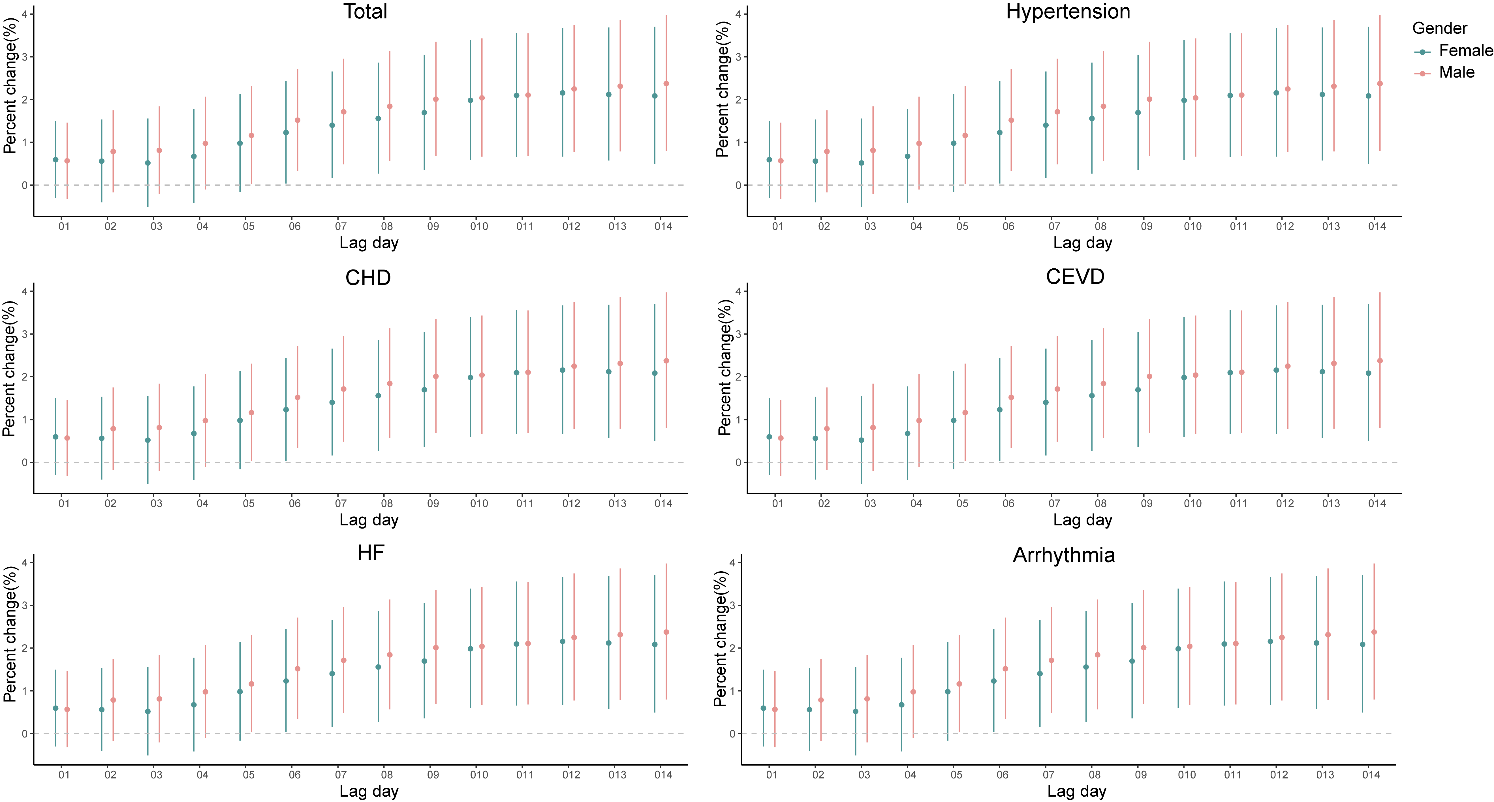


**Supplementary Figure 7. Percent changes and 95% CIs of daily hospital admissions for total and cause-specific circulatory system diseases by each 10 μg/m^3^ increase of PM_2.5_ concentrations stratified by gender in the cumulative-day lag models (dfs for secular time is 7 per year).**

Abbreviations: HBP, hypertension; CHD, Coronary Heart Disease; CEVD, Cerebrovascular Disease; HF, Heart Failure.


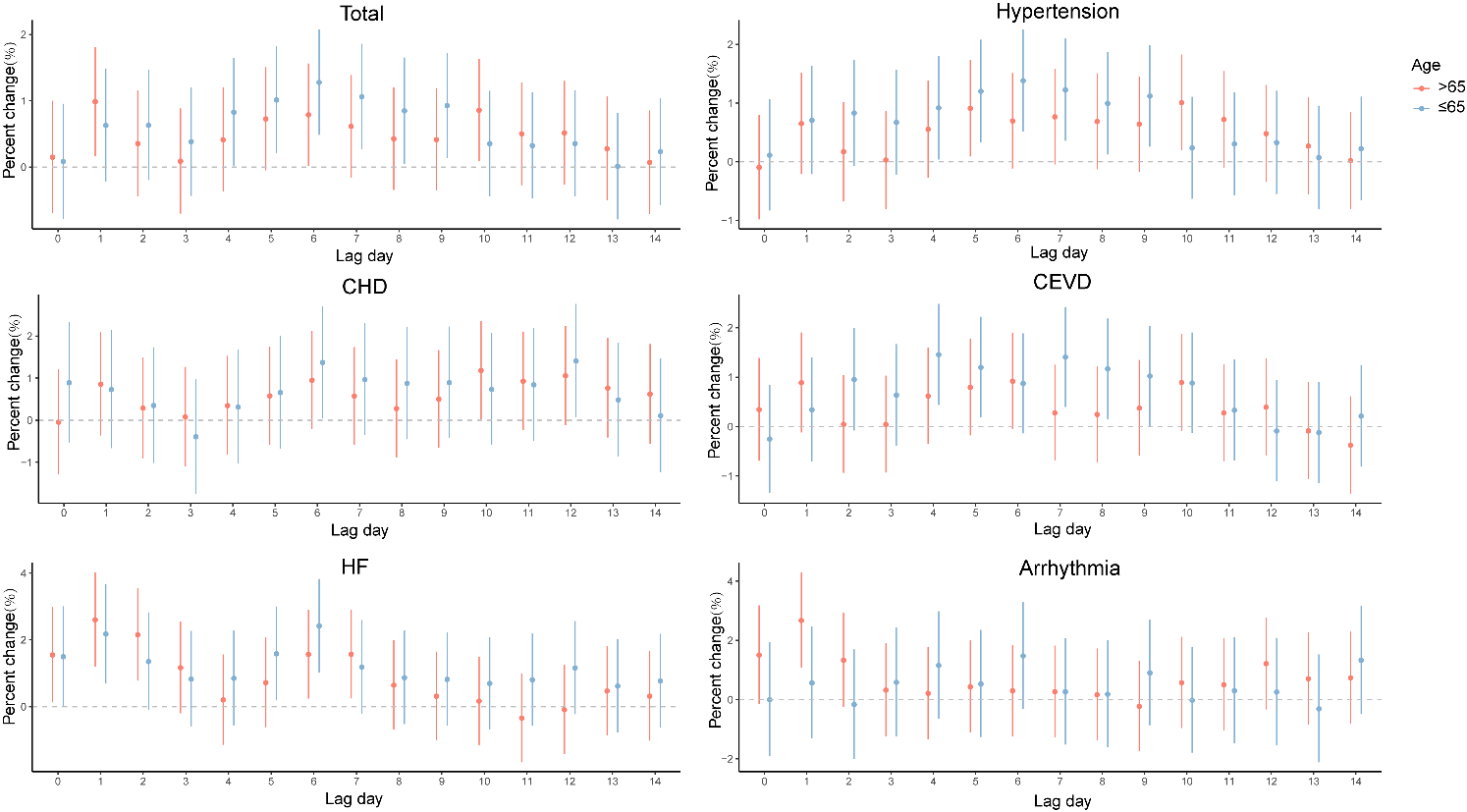


**Supplementary Figure 8. Percent changes and 95% CIs of daily hospital admissions for total and cause-specific circulatory system diseases by each 10 μg/m^3^ increase of PM_2.5_ concentrations stratified by age in the single-day lag models (dfs for secular time is 7 per year).**

Abbreviations: HBP, hypertension; CHD, Coronary Heart Disease; CEVD, Cerebrovascular Disease; HF, Heart Failure.


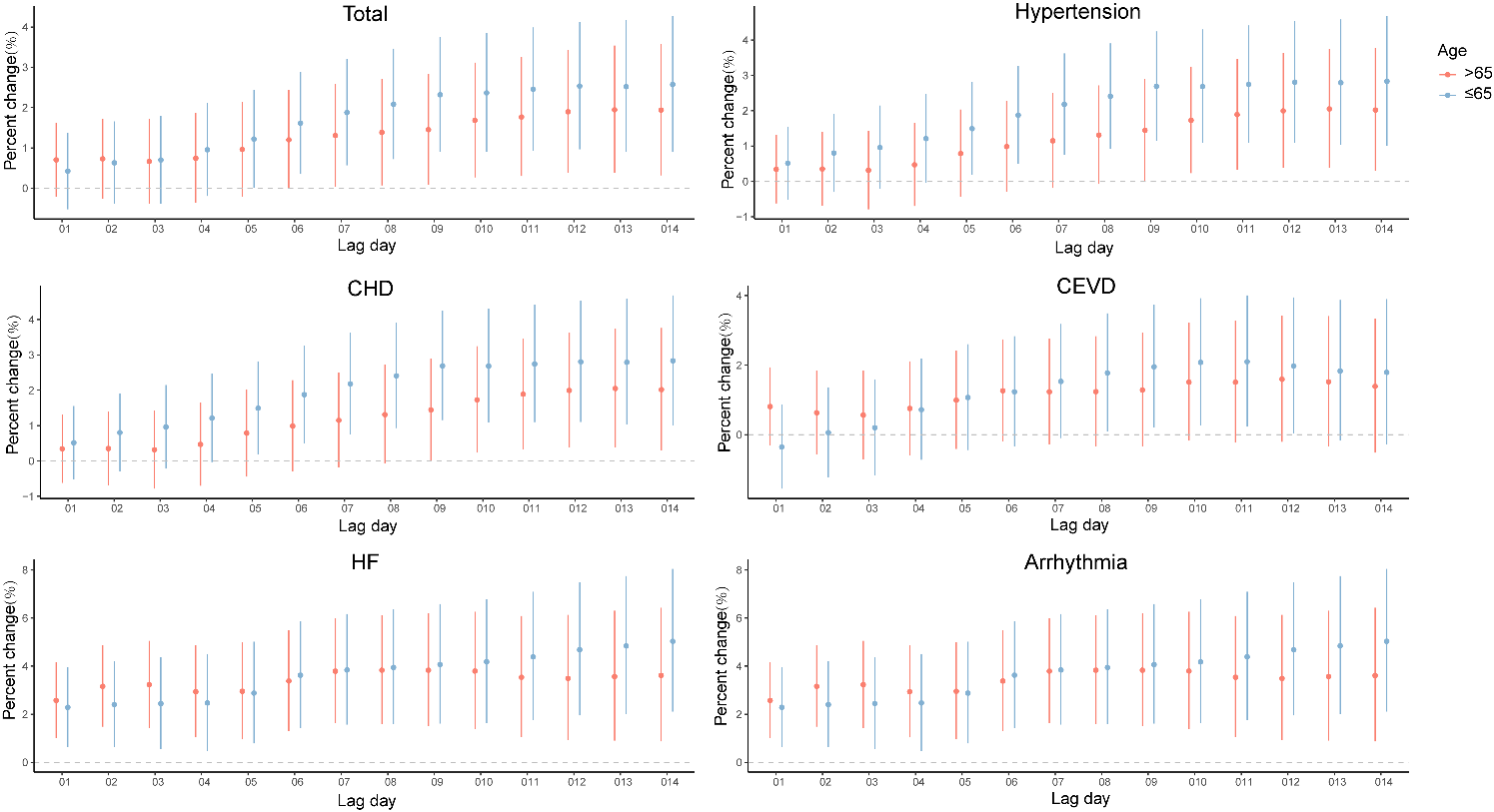


**Supplementary Figure 9. Percent changes and 95% CIs of daily hospital admissions for total and cause-specific circulatory system diseases by each 10 μg/m^3^ increase of PM_2.5_ concentrations stratified by age in the cumulative-day lag models (dfs for secular time is 7 per year).**

Abbreviations: HBP, hypertension; CHD, Coronary Heart Disease; CEVD, Cerebrovascular Disease; HF, Heart Failure.


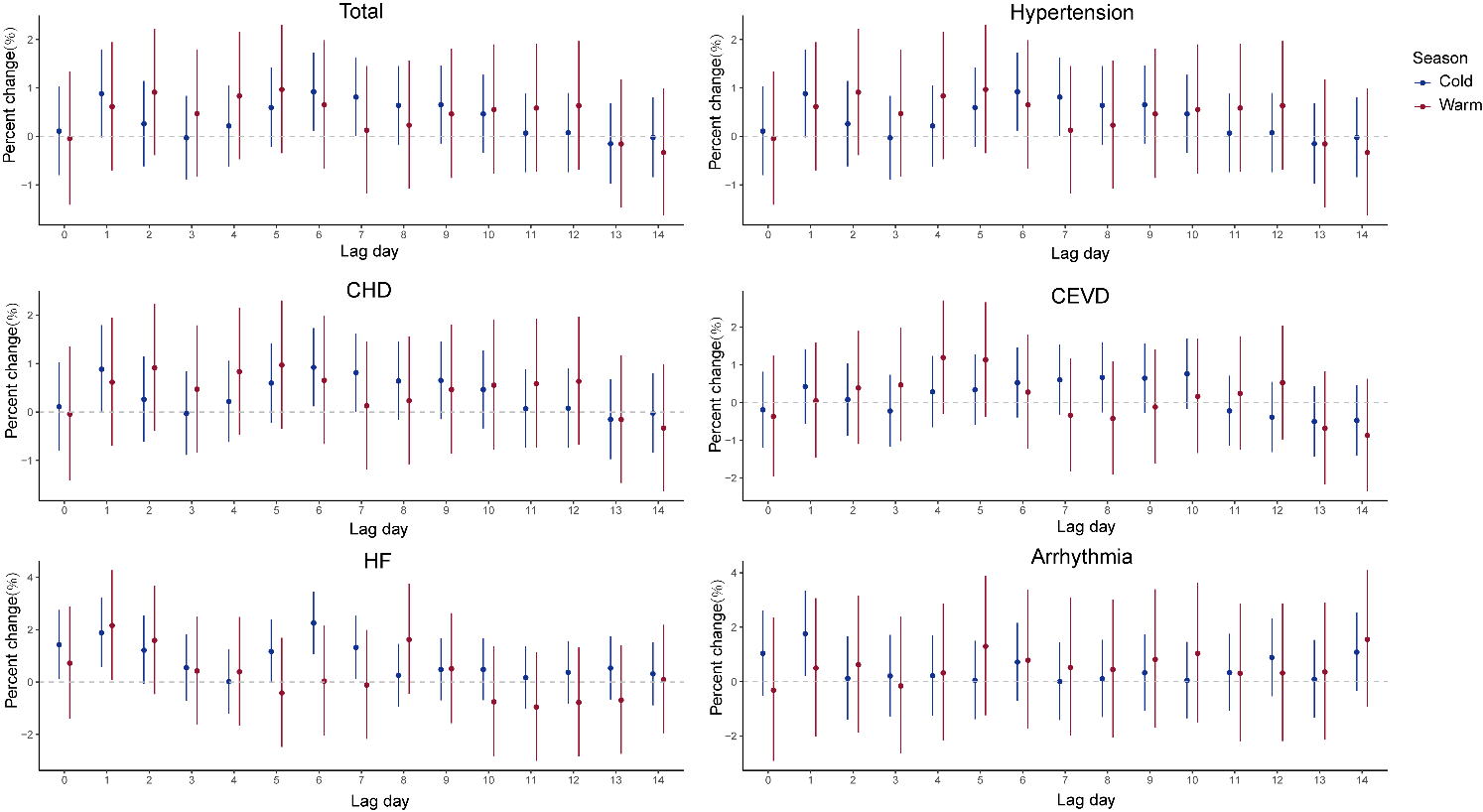


**Supplementary Figure 10. Percent changes and 95% CIs of daily hospital admissions for total and cause-specific circulatory system diseases by each 10 μg/m^3^ increase of PM_2.5_ concentrations stratified by season in the single-day lag models (dfs for secular time is 7 per year).**

Abbreviations: HBP, hypertension; CHD, Coronary Heart Disease; CEVD, Cerebrovascular Disease; HF, Heart Failure.


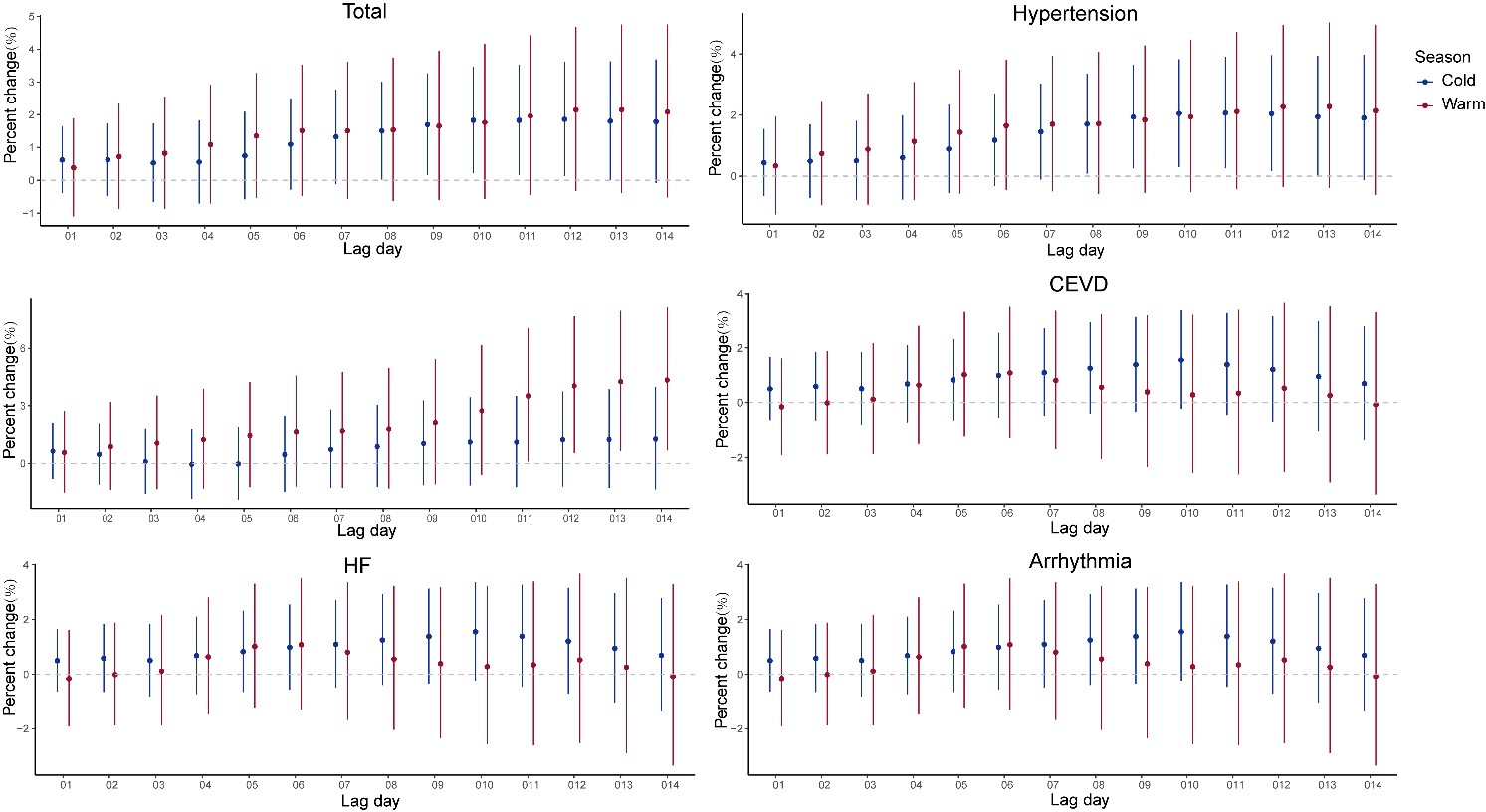


**Supplementary Figure 11. Percent changes and 95% CIs of daily hospital admissions for total and cause-specific circulatory system diseases by each 10 μg/m^3^ increase of PM_2.5_ concentrations stratified by season in the cumulative-day lag models (dfs for secular time is 7 per year).**

Abbreviations: HBP, hypertension; CHD, Coronary Heart Disease; CEVD, Cerebrovascular Disease; HF, Heart Failure.
